# Cardiolipin-Based Electrochemical Biosensor for Non-treponemal Antibody Detection Using Screen-Printed Electrodes: A Proof-of-Concept Study

**DOI:** 10.1021/acsomega.6c07930

**Published:** 2026-10-27

**Authors:** Duane da Silva Moraes, Bruna Ferri Serafini, Felipe Longaray Kadel, Gabriela Victória de Mello Jantzch, Milleny Germann Souza, Willyan Hasenkamp Carreira, Antonio Shigueaki Takimi, Julia Konzen Moreira

**Affiliations:** † Biosens Development and Industry of Biosensors LTDA (BIOSENS), São Leopoldo, RS 93022-715, Brazil; ‡ Postgraduate Program in Mining, Metallurgical and Materials Engineering, 28124 Federal University of Rio Grande do Sul (UFRGS), Porto Alegre, RS 91501-970, Brazil; § Postgraduate Program in Collective Health, University of Vale do Rio dos Sinos (UNISINOS), São Leopoldo, RS 93022-000, Brazil; ∥ Graduate Program of Materials Engineering, Federal University of Rio Grande do Sul (UFRGS), Porto Alegre, RS 91501-970, Brazil; ⊥ Postgraduate Program in Environmental Quality, 125099 Feevale University, Novo Hamburgo, RS 93525-075, Brazil; # Postgraduate Program in Chemistry, Federal University of Rio Grande do Sul (UFRGS), Porto Alegre, RS 91540-000, Brazil; ∇ Postgraduate Program in Bioengineering and Biotechnology, Swiss Federal Institute of Technology Lausanne (EPFL), Lausanne 1015, Switzerland

## Abstract

Syphilis remains a major public health concern. Although non-treponemal tests are widely used for screening and treatment monitoring, their interpretation relies on visual assessment of flocculation. Here, an amperometric biosensor using isolated cardiolipin (0.03–0.12%) as the sole biorecognition element was developed on carbon/graphene screen-printed electrodes. The 0.06% concentration was selected for subsequent experiments, and the biosensor discriminated positive and negative control materials across two electrode batches. Progressive positive-control dilution produced a systematic electrochemical response (*R* ^2^ = 0.921). Electrode functionalization significantly affected the response (*p* < 0.001), supporting the contribution of immobilized cardiolipin. Discrimination was maintained under physiological and pathological glucose conditions (adjusted *p* < 0.001). These findings provide a preliminary proof-of-concept for cardiolipin-based electrochemical assessment of non-treponemal antibody reactivity. Further studies using clinical samples are required to establish analytical and clinical performance and investigate semiquantitative evaluation by correlating electrochemical responses with defined serological titers.

## Graphical abstract

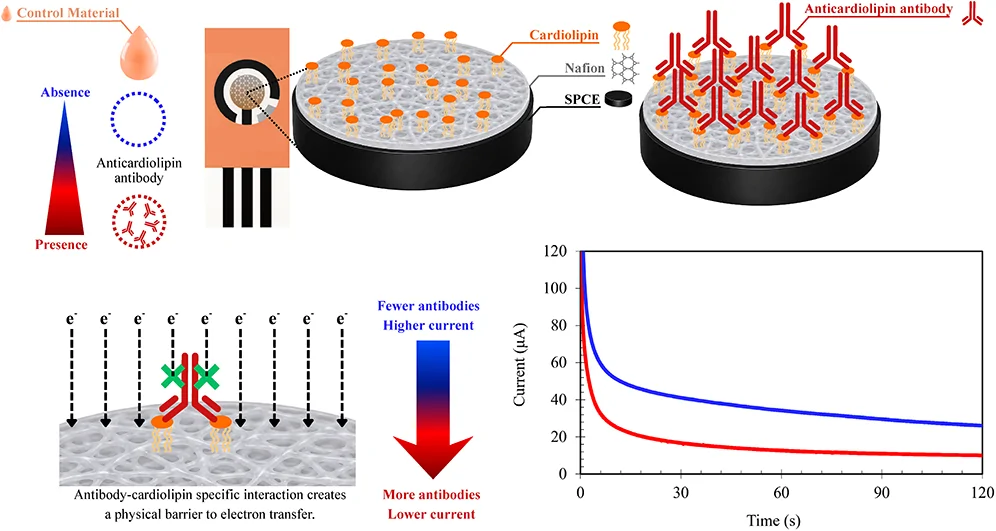

## 1 Introduction

Syphilis is a sexually transmitted infection caused by the spirochete *Treponema pallidum* (*T. pallidum*) and remains an important public health concern, particularly during pregnancy. Untreated maternal infection can result in vertical transmission and congenital syphilis, leading to adverse outcomes including miscarriage, preterm birth, stillbirth, with approximately 30% of untreated pregnancies resulting in fetal loss or death shortly after birth.^1–5^ Although syphilis is preventable and treatable, timely diagnosis and appropriate treatment remain essential for reducing transmission and improving maternal and neonatal outcomes. Therefore, diagnostic approaches that facilitate rapid and reliable detection and enable monitoring of treatment response remain clinically relevant, particularly in settings where access to centralized laboratory testing is limited.

Syphilis diagnosis generally relies on the complementary use of treponemal (TT) and non-treponemal tests (NTT). TTs detect antibodies directed against *T. pallidum* proteins and are theoretically highly specific.^1^ NTTs, such as the Rapid Plasma Reagin (RPR) and the Venereal Disease Research Laboratory (VDRL) tests, detect antibodies directed against lipid antigens containing cardiolipin, lecithin, and cholesterol. In addition to supporting the diagnosis of active infection, NTTs provide antibody titers that are clinically useful for monitoring treatment response through changes in antibody levels over time.^6^ However, conventional NTTs are based on flocculation reactions that require a secondary aggregation process and microscopic interpretation by trained personnel, introducing potential operator-dependent variability and limiting their decentralization. Commercial rapid tests have improved access to syphilis diagnosis but are predominantly qualitative and therefore do not provide the semiquantitative information required for treatment follow-up.^7,8^ These limitations highlight the need for simple analytical platforms capable of detecting non-treponemal antibodies while providing an objective and potentially semiquantitative response.

Electrochemical biosensors represent a promising approach for addressing these limitations because molecular recognition events at the electrode interface can be translated into measurable electrical signals. However, current literature reveals a scarcity of point-of-care devices specifically designed for the detection of non-treponemal antibodies in syphilis. Do Egito et al.^9^ reported a biosensor based on a gold electrode modified with self-assembled layers for immobilization of the RPR reagent, a colloidal suspension containing cardiolipin, cholesterol, and lecithin. Using cyclic voltammetry (CV) and electrochemical impedance spectroscopy (EIS), the authors demonstrated detection and semiquantification of anticardiolipin antibodies at different serological titers. Although this study demonstrated the feasibility of electrochemical detection of non-treponemal antibodies, the use of a complex RPR formulation as the recognition element and a gold electrode introduces additional considerations regarding antigen composition and platform cost. Furthermore, alternative electrochemical approaches, such as chronoamperometry, may provide a simpler measurement strategy for integration into portable sensing platforms.

In this context, the present study aimed to develop and evaluate an amperometric biosensor for the detection of non-treponemal antibodies associated with syphilis using isolated cardiolipin as the sole biorecognition element immobilized on carbon/graphene screen-printed electrodes. Unlike conventional NTTs and the previously reported electrochemical platform based on the complete RPR formulation,^9^ the proposed approach uses a chemically defined antigenic component without cholesterol or lecithin. The biosensor was evaluated for qualitative discrimination between positive and negative control materials and for its electrochemical response to progressive dilution of the positive control material. In addition, the contribution of immobilized cardiolipin to the electrochemical response was investigated by comparison with electrodes without cardiolipin, and the potential influence of an electroactive interferent was preliminarily assessed using glucose at physiological and pathological concentrations. This strategy combines a simplified recognition interface with a low-cost, screen-printed electrochemical platform and provides a preliminary proof-of-concept for the feasibility of this approach toward future point-of-care detection and monitoring of syphilis.

## 2 Materials and Methods

The experimental workflow comprised the preparation of antigenic solutions containing different cardiolipin concentrations and their immobilization onto screen-printed electrodes in combination with a polymeric coating. Subsequently, the electrochemical characteristics of the modified electrode interfaces were evaluated by cyclic voltammetry and electrochemical impedance spectroscopy, followed by qualitative evaluation of the antigen–antibody interaction using chronoamperometry to determine the most suitable cardiolipin concentration. The interaction between cardiolipin and non-treponemal antibodies was also investigated by microscopic evaluation. After selecting the immobilization condition, the effect of progressive dilution of the positive control material on the electrochemical response was evaluated. Finally, the contribution of immobilized cardiolipin to the electrochemical response and the potential interfering effect of glucose were investigated. A schematic representation of the experimental workflow is shown in Figure 1.

**Figure 1:**
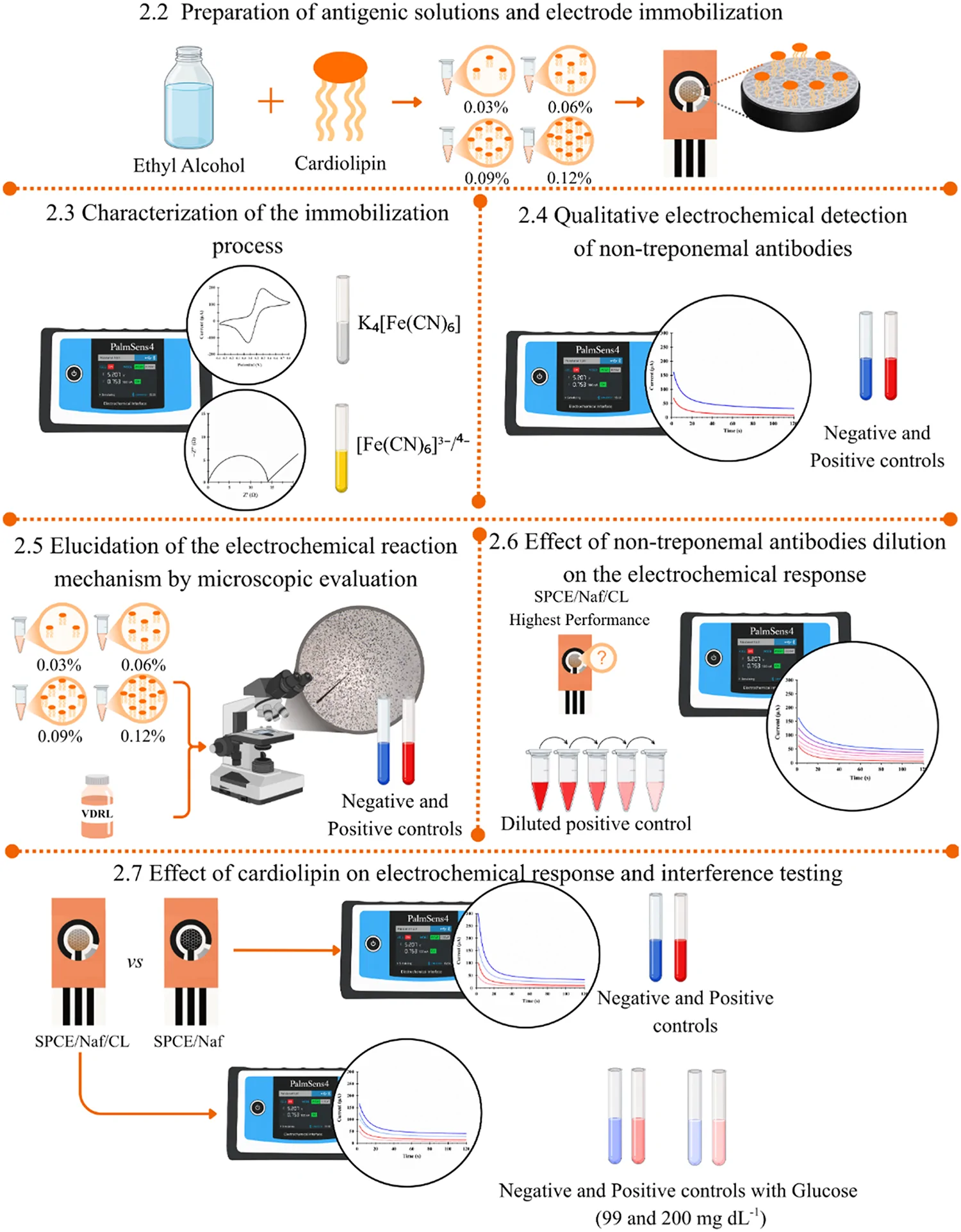
Schematic representation of methodology. SPCE/Naf/CL corresponds to the electrode modified with Nafion and immobilized cardiolipin. SPCE/Naf corresponds to the electrode modified with Nafion only. Selected graphical elements in this figure were generated using ChatGPT (OpenAI) and subsequently incorporated and edited by the authors.

### 2.1 Materials

14:0 Cardiolipin ammonium salt (1′,3′-bis­[1,2-dimyristoyl-*sn*-glycero-3-phosphoglycerol]) was purchased from Avanti Polar Lipids and employed as the antigenic recognition element. Nafion 117 solution (5%, Sigma-Aldrich) was used as the immobilization polymer. Ethanol (95%, Química Moderna) was used for cardiolipin resuspension.

Positive (Lot 621520) and negative (Lot 626730) control materials, as well as the antigen suspension containing cardiolipin, cholesterol, and lecithin, were obtained from a commercial VDRL kit (Wiener Diagnóstica, Manufacturer’s catalog number 1853155, Lot 2501626860). According to the manufacturer’s instructions, the positive and negative controls consist of dilutions of inactivated serum tested and found nonreactive for hepatitis B surface antigen, hepatitis C virus, and human immunodeficiency virus. The manufacturer does not explicitly specify whether the serum is of human or animal origin and classifies the controls as reactive and nonreactive, respectively, but does not provide an assigned VDRL titer. The detailed composition of the control matrix and the presence or identity of preservatives are not specified in the manufacturer’s documentation. Therefore, the VDRL titer of 1:8 reported in this study for the positive control was not manufacturer-assigned but was experimentally determined in our laboratory using the conventional VDRL titration procedure described in Section 2.6.

Potassium ferrocyanide (Labsynth), potassium ferricyanide (Cinética), and potassium chloride (Química Moderna) were used for electrochemical characterization, while anhydrous d-glucose (Labsynth) was used for interference testing.

The electrodes were supplied by Biosens Desenvolvimento e Indústria de Biossensores LTDA and consisted of a three-electrode system fabricated by screen-printing technology. The working and counter electrodes were fabricated from a carbon/graphene composite conductive paste, while the reference electrode was composed of silver/silver chloride (Ag/AgCl). In addition, the electrodes incorporated a black vinyl spacer with an 8 mm diameter to define the reaction area and a transparent vinyl spacer with a 4 mm diameter positioned over the working electrode to delimit the reagent immobilization area.

Two independently prepared electrode batches were used throughout the study. One electrode batch was used for the qualitative evaluation of different cardiolipin concentrations. A second, independently prepared batch was used for the serial dilution experiments with control materials, evaluation of the effect of cardiolipin immobilization, and interference studies.

### 2.2 Preparation of Antigenic Solutions and Electrode Immobilization

The proposed methodology is illustrated in Figure 2 and was developed based on the adaptation and optimization of a previously patented technology (BR1020250081946).

**Figure 2:**
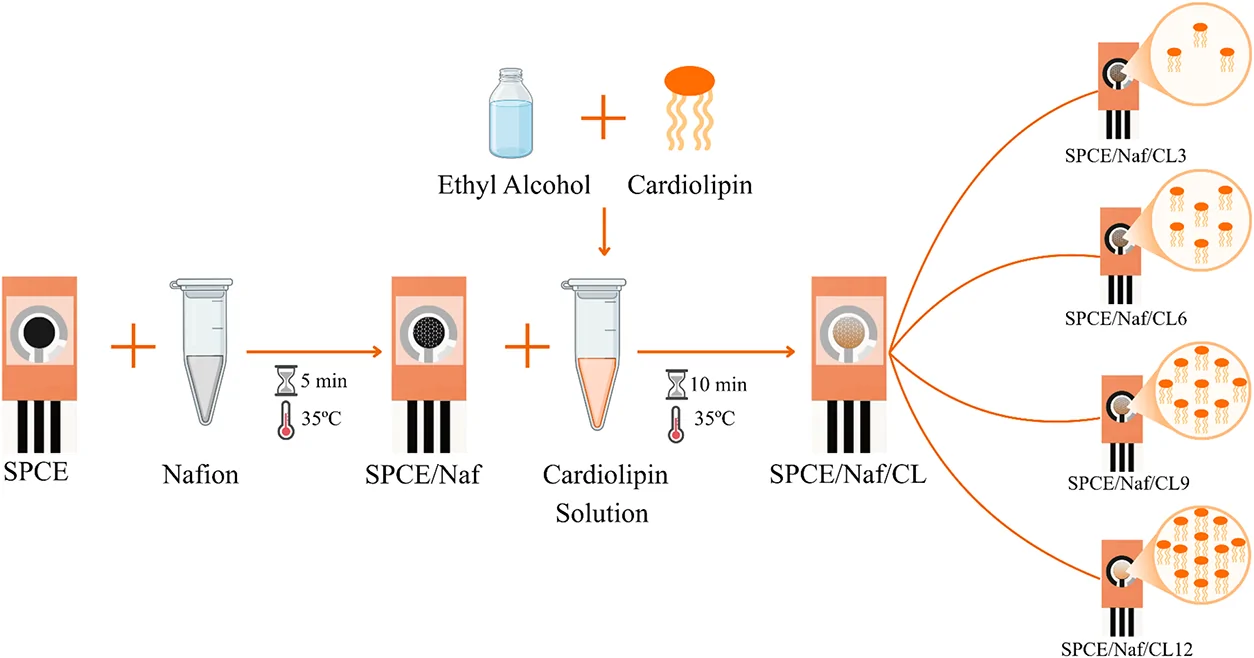
Schematic representation of the immobilization process of screen-printed carbon/graphene electrodes with Nafion and cardiolipin. SPCE corresponds to the unmodified electrode; SPCE/Naf refers to the Nafion-modified electrode; SPCE/Naf/CL corresponds to the electrode modified with Nafion and immobilized cardiolipin. SPCE/Naf/CL3, SPCE/Naf/CL6, SPCE/Naf/CL9, and SPCE/Naf/CL12 correspond to biosensors functionalized with cardiolipin solutions at concentrations of 0.03%, 0.06%, 0.09%, and 0.12% (w/v), respectively.

First, 5 μL of Nafion solution was deposited onto the working electrode and dried at 35 °C for 5 min to form a polymeric film. This step was intended to generate a stable and uniform support layer capable of promoting cardiolipin adhesion to the electrode surface and minimizing its leaching during subsequent experimental procedures. In addition, Nafion has been widely employed in electrochemical sensors due to its ability to provide a favorable microenvironment for biomolecular interactions, contributing to improved stability, sensitivity, and reproducibility of the sensing platform.^10,11^

Cardiolipin solutions at different concentrations were prepared to evaluate, in an immobilized configuration on screen-printed electrodes, their ability to interact with non-treponemal antibodies associated with syphilis. Four antigenic solutions were prepared by dissolving cardiolipin in 1 mL of 95% ethanol to obtain final concentrations of 0.03%, 0.06%, 0.09%, and 0.12% (w/v). Subsequently, 5 μL of the cardiolipin solution at the desired concentration was deposited onto the Nafion-coated working electrode and dried at 35 °C for 10 min to allow formation of the antigenic layer. The functionalized electrodes were then stored at room temperature in a desiccator until use.

### 2.3 Characterization of the Immobilization Process

To characterize the immobilization steps and evaluate the impact of the reagents employed and their respective concentrations, CV and EIS were performed on unmodified electrodes (SPCE), electrodes modified only with Nafion (SPCE/Naf), and electrodes modified with Nafion and cardiolipin at each antigen solution concentration (SPCE/Naf/CL3, SPCE/Naf/CL6, SPCE/Naf/CL9, and SPCE/Naf/CL12).

CV were carried out using a PalmSens4 portable potentiostat controlled by PSTrace software (version 5.8). Measurements were performed over a potential range from +0.8 to −0.4 V at a scan rate of 0.1 V s^–1^, comprising 10 consecutive cycles per assay. All experiments were conducted in triplicate for each experimental condition. A 20 μL aliquot of 10 mM potassium ferrocyanide (K_4_[Fe­(CN)_6_]) prepared in 1 M potassium chloride (KCl) was used as the redox probe.

For data analysis, the cathodic peak current (*i*_pc_) and anodic peak current (*i*_pa_) were extracted from the fifth voltammetric cycle, as these parameters provide information regarding electron transfer at the electrode/solution interface. Subsequently, the peak-to-peak potential separation (Δ*E*), defined as the difference between anodic (*E*_pa_) and cathodic (*E*_pc_) peak potentials (Δ*E* = *E*_pa_ – *E*_pc_), was calculated as an indicator of the reversibility of the redox system.

The electrochemical symmetry of the system was assessed through the absolute value of the peak current ratio (|*i*_pa_/*i*_pc_|). Values close to unity indicate a reversible and symmetric electrochemical behavior, whereas deviations may be associated with diffusion limitations, electrode surface heterogeneities, or effects arising from surface modification processes.^12–14^

EIS were also performed using the PalmSens4 potentiostat controlled by PSTrace software (version 5.8). A 20 μL aliquot of 10 mM potassium ferri/ferrocyanide ([Fe­(CN)_6_]^3–/4–^, 1:1 molar ratio) prepared in 1 M KCl was used as the redox probe. Prior to each EIS measurement, the open-circuit potential (OCP) was monitored for 10 s, and the impedance spectrum was subsequently recorded at the measured OCP using an AC potential amplitude of 0.1 V over a frequency range from 0.01 Hz to 100 kHz, with 5 points per decade. EIS measurements were performed in triplicate for each experimental condition. The resulting impedance spectra were fitted using the Randles equivalent circuit, and the charge-transfer resistance (*R*_ct_) was extracted to evaluate changes in the sensor/electrolyte interface associated with the successive immobilization steps.^15,16^

### 2.4 Qualitative Electrochemical Detection of Non-treponemal Antibodies

Initially, the interaction between the immobilized cardiolipin and control materials containing or lacking non-treponemal antibodies was qualitatively evaluated by chronoamperometry. This technique was selected to monitor changes in the electrochemical response associated with modifications of the electrode/electrolyte interface following antigen–antibody recognition. Previous electrochemical studies have shown that the formation of antigen–antibody complexes at modified electrode surfaces can alter interfacial properties and consequently modulate the measured electrical current.^9^

Chronoamperometric measurements were performed using a PalmSens4 portable potentiostat controlled by PSTrace software (version 5.8). A constant potential of +0.6 V was applied for 120 s, based on a previously established chronoamperometric protocol using the same carbon/graphene screen-printed electrode platform.^17^ No exogenous redox probe or supporting electrolyte was added during these measurements; 25 μL of either positive or negative commercial control material was deposited directly onto the sensing area defined by the 8 mm vinyl well, ensuring complete coverage of the working, reference, and counter electrodes.

Three consecutive chronoamperometric measurements were performed on each electrode, with a 3 min resting interval between measurements. This strategy was adopted to investigate the influence of the interaction time between the analyte and the bioreceptor, since the reference VDRL methodology performed in clinical laboratories includes a 4 min orbital agitation step before microscopic evaluation to allow formation of the antigen–antibody reaction product.^18^

Because no defined redox probe was present, the recorded current could not be unequivocally attributed to a specific faradaic process, and contributions from interfacial capacitive processes and electroactive components of the control matrix cannot be excluded under the present experimental conditions.

Each experimental condition, defined by the combination of cardiolipin concentration (0.03%, 0.06%, 0.09%, or 0.12%) and control material (positive or negative), was evaluated using four separate electrodes as technical replicates (*n* = 4 sensors per condition). The same commercial control material was used across the four technical replicates; therefore, these replicates do not represent independent biological samples. Three consecutive chronoamperometric measurements were performed on each electrode. Overall, the experimental design comprised 96 electrochemical measurements, corresponding to four cardiolipin concentrations, two commercial control materials, four electrodes per condition and three consecutive measurements per electrode. All experimental steps, including the cardiolipin immobilization procedure, were performed in a Class 100,000 (ISO 8) cleanroom under controlled environmental conditions, with a temperature of 20 ± 5 °C and relative humidity of 50 ± 5%. These conditions were maintained to minimize the influence of environmental variations on the electrode immobilization process, thereby improving experimental standardization and contributing to the stability of the immobilized cardiolipin throughout the assays.

For data analysis, the electrical current recorded at 5 s after potential application was extracted from each chronoamperogram. This time point was selected because it corresponds to the initial sloped region of the current–time curve, where current changes are more pronounced, thereby providing improved discrimination between positive and negative materials. The current values extracted from the first, second, and third measurements were designated as *i*_5,1_, *i*_5,2_, and *i*_5,3_, respectively. Descriptive statistical analysis, including calculation of the mean and standard deviation, was performed for each variable to evaluate the central tendency and data dispersion.

Current data were analyzed using a linear mixed-effects model to account for the repeated-measures structure of the experimental design, as three consecutive measurements (*i*_5,1_, *i*_5,2_, and *i*_5,3_) were performed on the same electrode. Thus, the three measurements obtained from the same electrode were not treated as independent observations.

The current recorded at 5 s was used as the dependent variable. Cardiolipin concentration (0.03%, 0.06%, 0.09%, and 0.12%), control material (positive and negative), and measurement number (*i*_5,1_, *i*_5,2_, and *i*_5,3_) were included in the model as fixed effects. The main effects of these factors, all two-way interactions, and the three-way interaction among cardiolipin concentration, control material, and measurement number were evaluated. Consecutive measurements performed on the same electrode were modeled as repeated measures using an unstructured covariance structure (UN) to account for within-electrode correlation.

Model parameters were estimated using restricted maximum likelihood (REML), and degrees of freedom were determined using the Kenward–Roger approximation. The significance of fixed effects was assessed using Type III F-tests, with a significance level of 5%. Given the significant interaction involving cardiolipin concentration, control material, and measurement number, comparisons between the positive and negative control materials were performed within each combination of cardiolipin concentration and measurement number. The resulting *p*-values were adjusted for multiple comparisons using the Holm procedure.

Model assumptions were assessed by visual inspection of the residuals. Approximate normality was evaluated using a quantile–quantile (Q–Q) plot, whereas the adequacy of the residual variance and the absence of systematic patterns were assessed using a residuals-versus-fitted-values plot. The residuals showed an approximately normal distribution, with some deviations in the tails, and no evident systematic pattern or pronounced heteroscedasticity was observed in the residuals-versus-fitted-values plot. Statistical analyses were performed using IBM SPSS Statistics with the Linear Mixed Models procedure.

### 2.5 Elucidation of the Electrochemical Reaction Mechanism by Microscopic Evaluation

To elucidate the reaction mechanism using cardiolipin in the absence of cholesterol and lecithin and to investigate whether the electrochemical response can be generated in the absence of the flocculation reaction, different cardiolipin concentrations were evaluated by optical microscopy. These conditions were compared with a commercial VDRL reagent containing cardiolipin associated with cholesterol and lecithin, which is routinely employed as a reference reagent for the microscopic detection of flocculation indicative of non-treponemal antibodies associated with syphilis.

Microscopic evaluation of the antigen–antibody interaction was performed according to the standardized VDRL protocol routinely used in clinical laboratories.^18^ Briefly, a Kline slide was prepared with individual reaction circles corresponding to different antigenic conditions: cardiolipin solutions without cholesterol and lecithin at concentrations of 0.03%, 0.06%, 0.09%, and 0.12% (w/v), and two reactions using the commercial VDRL reagent (gold standard), which contains cholesterol and lecithin. The cardiolipin solutions were evaluated in the presence of the positive control, whereas the commercial VDRL reagent was evaluated with both the positive and negative controls. Each reaction consisted of 20 μL of the respective antigenic solution mixed with 50 μL of the material control.

The slide was subjected to orbital shaking (KLINE-KJ201BS shaker, Laborcare) at 180 rpm for 4 min. Subsequently, the reactions were examined under an optical microscope (Primo Star, Zeiss) using 40× magnification by a trained laboratory professional to assess the presence of flocculation.

The presence of flocculation was interpreted as evidence of antigen–antibody complex formation followed by aggregation into microscopically detectable structures. In the conventional VDRL assay, this phenomenon occurs because reagin antibodies present in the control material bind multivalently to cardiolipin-lecithin-cholesterol antigenic particles, promoting the formation of larger immune complexes that produce the characteristic flocculation pattern.^19^ The reaction between the commercial VDRL reagent and the negative control was included as a control for the absence of flocculation. Conversely, the absence of flocculation with the isolated cardiolipin solutions was interpreted as indicating that no microscopically detectable aggregation occurred under the evaluated conditions, although molecular interactions between cardiolipin and antibodies may still occur without producing the characteristic flocculation observed with the complete VDRL antigen formulation.

### 2.6 Effect of Non-treponemal Antibodies Dilution on the Electrochemical Response

To evaluate the ability of the biosensor to detect different levels of non-treponemal antibodies, serial dilutions of the positive control were prepared using the negative control as the diluent. Initially, a 1:2 (v/v) dilution was obtained by mixing 180 μL of the positive control with 180 μL of the negative control. Subsequently, 180 μL aliquots of the resulting solution were sequentially transferred to new tubes containing 180 μL of the negative control, yielding progressive dilutions of 1:4, 1:8, and 1:16. The selected dilution factors were based on the VDRL titer of the positive control material, which was determined to be 1:8 according to the reference VDRL assay.

Electrochemical measurements were performed following the protocol described in Section 2.4. Electrodes functionalized with the cardiolipin concentration that exhibited the highest discriminatory capability in the previous experiments were employed. Likewise, the chronoamperometric measurement that provided the greatest discrimination between positive and negative materials was selected to evaluate the electrochemical response to progressive dilution of the positive control material.

For each experimental condition, four independent measurements were performed (*n* = 4). In each assay, 25 μL of control material was deposited onto the active sensing area, followed by the application of a constant potential of +0.6 V for 120 s using a PalmSens4 portable potentiostat controlled by PSTrace software (version 5.8). All subsequent experiments were conducted in a conventional laboratory environment without specific control of temperature or relative humidity. These experimental conditions were intentionally adopted to approximate the environmental conditions expected during device use in a real-world testing setting and to evaluate the performance of the platform outside strictly controlled environmental conditions.

The electrochemical response was quantified as the current measured 5 s after potential application. Results were expressed as mean ± standard deviation of the quadruplicate measurements. To investigate the relationship between the electrochemical response and the dilution factor of the positive control material, the mean current values were plotted against the log_2_-transformed dilution factor and subsequently analyzed by linear regression. The coefficient of determination (*R* ^2^) was used to assess the goodness-of-fit and the linearity of the biosensor response over the evaluated range of non-treponemal antibody dilutions.

### 2.7 Effect of Cardiolipin on Electrochemical Response and Interference Testing

To evaluate whether the discrimination between positive and negative controls was dependent on the presence of immobilized cardiolipin, electrodes modified with Nafion only (SPCE/Naf) were prepared following the same immobilization protocol described in Section 2.2 and subsequently evaluated according to the chronoamperometric protocol described in Section 2.6. Each experimental condition was evaluated using four separate electrodes as technical replicates (*n* = 4 electrodes per condition), with 25 μL of control material deposited onto each electrode. The electrochemical response was determined from the current recorded 5 s after potential application. Results were expressed as mean ± standard deviation and were analyzed using a two-way factorial analysis of variance (ANOVA), with control material (positive and negative) and electrode immobilization (SPCE/Naf/CL6 and SPCE/Naf) as fixed factors and the current recorded at 5 s as the dependent variable. The main effects of control material and electrode immobilization were evaluated using Type III sums of squares. Simple-effect analyses were subsequently performed to compare positive and negative control materials within each electrode immobilization and to compare electrode immobilization within each control material. Statistical significance was established at *p* < 0.05. Statistical analyses were performed using IBM SPSS Statistics with the General Linear Model (GLM) Univariate procedure.

Additionally, to assess the potential contribution of electroactive interfering species to the electrochemical response, glucose was selected as a potential interferent. Glucose is present in biological matrices and may exhibit an electrochemical response at potentials close to +0.6 V, as reported in electrochemical glucose detection studies, and may therefore contribute to the current measured under the conditions employed in this study.^20^ For the interference assay, glucose was added separately to 200 μL of the positive and negative control materials. Two glucose concentrations were evaluated: 99 mg dL^–1^, representing the upper limit of the normal fasting glucose range and defined herein as the physiological condition, and 200 mg dL^–1^, representing a markedly elevated glucose concentration and defined herein as the pathological condition.^21^

To evaluate the discrimination between the positive and negative controls in the presence of glucose, SPCE/Naf/CL6 electrodes were prepared following the same immobilization protocol described in Section 2.2 and subsequently evaluated according to the chronoamperometric protocol described in Section 2.6. Each experimental condition was evaluated using four separate electrodes as technical replicates (*n* = 4 electrodes per condition), with 25 μL of control material deposited onto each electrode. The electrochemical response was determined from the current recorded 5 s after potential application. Results were expressed as mean ± standard deviation and were analyzed using a two-way factorial analysis of variance (ANOVA), with control material (positive and negative) and glucose condition (without interferent, physiological, and pathological) as fixed factors and the current recorded at 5 s as the dependent variable. The main effects of control material and glucose condition were evaluated using Type III sums of squares. Pairwise comparisons between control materials within each glucose condition and among glucose conditions within each control material were performed with Bonferroni adjustment for multiple comparisons. Statistical significance was established at *p* < 0.05. Statistical analyses were performed using IBM SPSS Statistics with the General Linear Model (GLM) Univariate procedure.

## 3 Results

### 3.1 Characterization of the Immobilization Process

Figure 3 presents the electrochemical characterization of the electrode immobilization process. The selection of the fifth cycle for presentation and analysis of the CV results was based on preliminary analyses presented in the Supporting Information (Figure S1). For all evaluated conditions, including the unmodified electrodes, Nafion-modified electrodes, and electrodes modified with Nafion and different cardiolipin concentrations, a progressive increase in current was observed with increasing cycle number. However, the variation between consecutive cycles remained below 1 μA, indicating only minor changes in the voltammetric response throughout the ten cycles. Therefore, the fifth cycle was selected as an intermediate and representative point within the sequence, avoiding reliance on either the initial or final cycles, and providing a representative view of the electrochemical behavior observed throughout the analysis.

**Figure 3:**
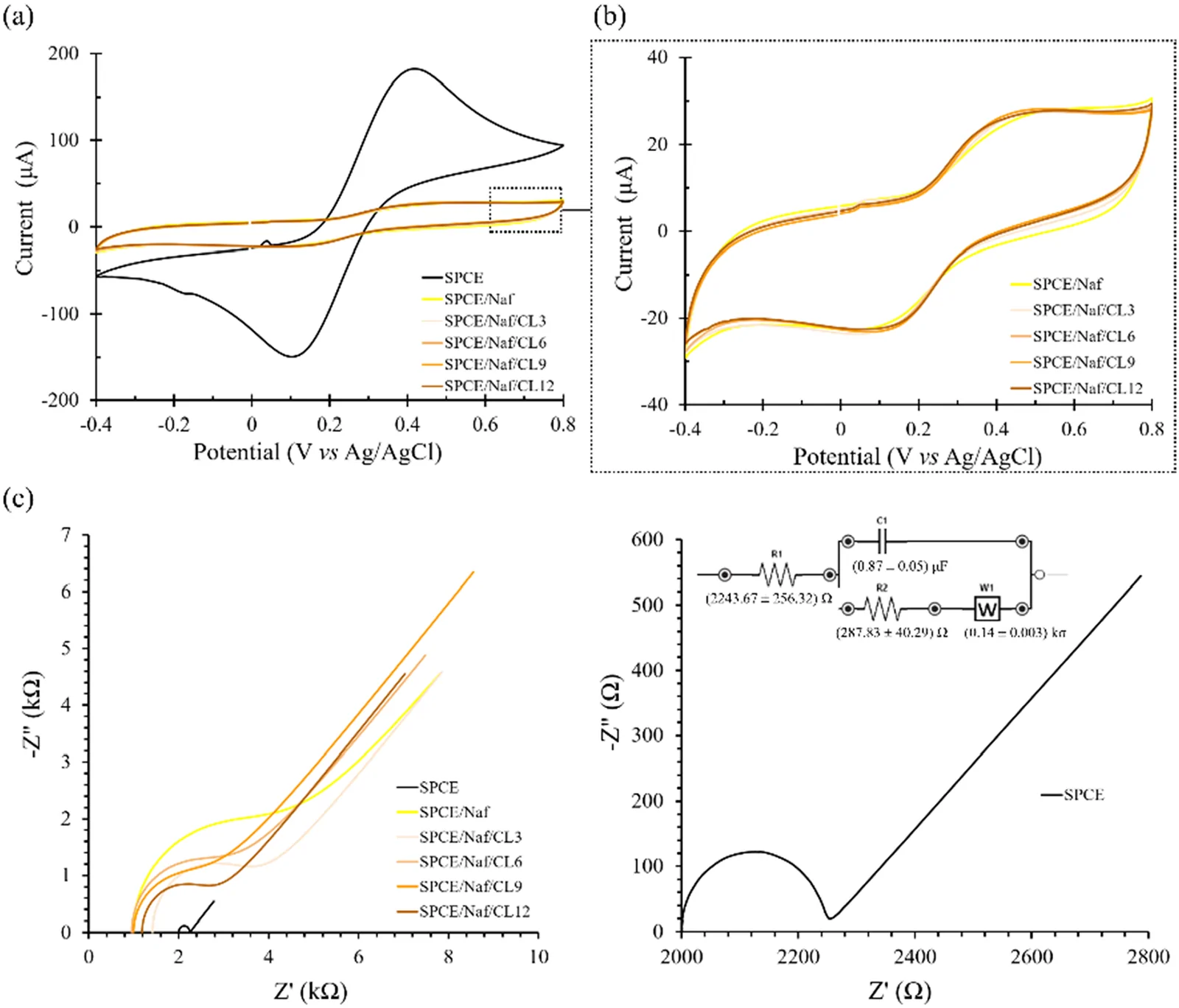
Electrochemical characterization of the different electrode modification stages: SPCE represented in black, SPCE/Naf represented in yellow, and SPCE/Naf/CL3, SPCE/Naf/CL6, SPCE/Naf/CL9, and SPCE/Naf/CL12 represented by an orange color gradient from the lightest to the darkest shade with increasing cardiolipin concentration. (a) Average cyclic voltammograms of the fifth cycle obtained in 10 mM K_4_[Fe­(CN)_6_] prepared in 1 M KCl. (b) Magnified view highlighting the voltammetric profiles from SPCE/Naf to SPCE/Naf/CL12. (c) Average electrochemical impedance spectra fitted using the Randles equivalent circuit, with the Nyquist plot for SPCE shown separately to improve visualization. All measurements were performed in triplicate.

The electrochemical parameters extracted from the voltammograms, including the *i*_pa_, *i*_pc_, Δ*E*, and |ipa/ipc|, together with the *R*_ct_ obtained from the electrochemical impedance spectra, are summarized in Table 1.

**Table 1:** Electrochemical Parameters Obtained from CV and EIS at Each Stage of the Electrode Immobilization Process^a^

Parameter	*i*_pa_ (μA)	*i*_pc_ (μA)	Δ*E* (mV)	|*i*_pa_/*i*_pc_|	*R*_ct_ (Ω)
SPCE	133.65 ± 41.85	–149.61 ± 6.21	303.33 ± 35.12	0.90 ± 0.29	287.83 ± 40.29
SPCE/Naf	24.58 ± 2.76	–21.99 ± 0.68	326.67 ± 90.18	1.12 ± 0.09	2716.50 ± 260.92
SPCE/Naf/CL3	27.01 ± 2.33	–23.12 ± 1.85	350.00 ± 26.46	1.17 ± 0.03	1758.50 ± 361.33
SPCE/Naf/CL6	25.97 ± 1.56	–21.92 ± 1.34	296.67 ± 28.87	1.19 ± 0.09	1807.50 ± 177.48
SPCE/Naf/CL9	27.09 ± 4.35	–22.57 ± 3.34	306.67 ± 28.87	1.20 ± 0.02	1416.00 ± 89.10
SPCE/Naf/CL12	23.37 ± 6.07	–22.30 ± 2.16	296.67 ± 86.22	1.04 ± 0.24	1560.50 ± 241.12

a Data are presented as mean ± standard deviation from triplicate measurements (*n* = 3).

Based on the results, the SPCE exhibited well-defined anodic (133.65 ± 41.85 μA) and cathodic (−149.61 ± 6.21 μA) peak currents, indicating efficient electron transfer of the redox probe at the electrode/electrolyte interface. The Δ*E* value (303.33 ± 35.12 mV), together with the peak current ratio close to unity (|*i*_pa_/*i*_pc_| = 0.90 ± 0.29), indicates a stable electrochemical response, consistent with that expected for screen-printed carbon-based electrodes employed in electrochemical sensing applications.^17,22^ Consistent with the voltammetric response, EIS analysis indicated a relatively low *R*_ct_ at the unmodified electrode/electrolyte interface (287.83 ± 40.29 Ω), supporting more favorable electron-transfer kinetics compared with the subsequently modified electrodes.^23,24^ These results demonstrate that the unmodified electrode provides a suitable electrochemical baseline for evaluating the effects of the subsequent surface modification steps.

Following modification of the working electrode with Nafion, a marked decrease in both the anodic (24.58 ± 2.76 μA) and cathodic (−21.99 ± 0.68 μA) peak currents was observed, accompanied by a slight increase in the Δ*E* value (326.67 ± 90.18 mV) and a pronounced increase in *R*_ct_ (2716.50 ± 260.92 Ω). These complementary CV and EIS results indicate that deposition of the polymeric film creates a barrier to charge transport and electron transfer at the electrode/electrolyte interface. Such an effect is expected for Nafion films, as the polymer possesses an anionic structure and ion-selective properties that can restrict the diffusion and mobility of electroactive species at the electrode/electrolyte interface.^25,26^

Modification of the electrodes with different cardiolipin concentrations did not produce substantial changes in the voltammetric profiles, shown in greater detail in Figure 3b, compared with the SPCE/Naf, and no significant differences were observed among the cardiolipin concentrations evaluated. As summarized in Table 1, the anodic peak currents ranged from 23.37 ± 6.07 μA to 27.09 ± 4.35 μA, whereas the cathodic peak currents varied from −21.92 ± 1.34 μA to −23.12 ± 1.85 μA, remaining comparable to those obtained for SPCE/Naf. Similarly, Δ*E* values showed only minor variations across the different conditions, ranging from 296.67 ± 28.87 mV to 350.00 ± 26.46 mV. These findings indicate that the major modulation of the voltammetric response occurred after deposition of the Nafion film, whereas subsequent cardiolipin immobilization produced only minor changes in the CV parameters.

Similar behavior has been reported for lipid film-modified electrodes, in which the preservation of peak currents and redox potentials close to those of the previously functionalized electrode indicates that the biological layer does not completely block electron transfer, suggesting either partial surface coverage or the formation of films containing regions permeable to the diffusion of electroactive species.^27^

Furthermore, the symmetry ratios remained close to unity under all experimental conditions, ranging from 1.04 ± 0.24 to 1.20 ± 0.02, indicating relatively symmetrical oxidation and reduction responses. However, considering the observed peak separation, the electrochemical behavior of the cardiolipin-functionalized electrode is more appropriately described as quasi-reversible.

EIS further revealed changes in the electrode/electrolyte interface following cardiolipin incorporation that were less evident from the voltammetric parameters. While Nafion deposition markedly increased the charge-transfer resistance relative to the unmodified electrode, subsequent incorporation of cardiolipin resulted in lower *R*_ct_ values. Among the cardiolipin-modified electrodes, *R*_ct_ ranged from 1416.00 ± 89.10 Ω to 1807.50 ± 177.48 Ω, with no significant differences among the cardiolipin concentrations evaluated. Thus, cardiolipin incorporation modified the interfacial electrochemical properties relative to SPCE/Naf, while variation in cardiolipin concentration produced comparatively minor changes in charge-transfer resistance. These findings complement the CV results and provide additional electrochemical evidence of surface modification associated with cardiolipin incorporation.

Taken together, the CV and EIS results indicate that Nafion deposition produced the most pronounced change in the electrochemical behavior relative to the unmodified SPCE, whereas subsequent cardiolipin incorporation further modified the electrode/electrolyte interface without substantially impairing electron transfer. The preservation of electron-transfer capability following cardiolipin immobilization is desirable, as it indicates that biological functionalization modified the electrode interface without compromising its electrochemical activity.^28^

### 3.2 Qualitative Electrochemical Detection of Non-treponemal Antibodies

Figure 4 illustrates the electrochemical performance of biosensors functionalized with different cardiolipin concentrations for discriminating between positive and negative control materials across the three consecutive measurements.

**Figure 4:**
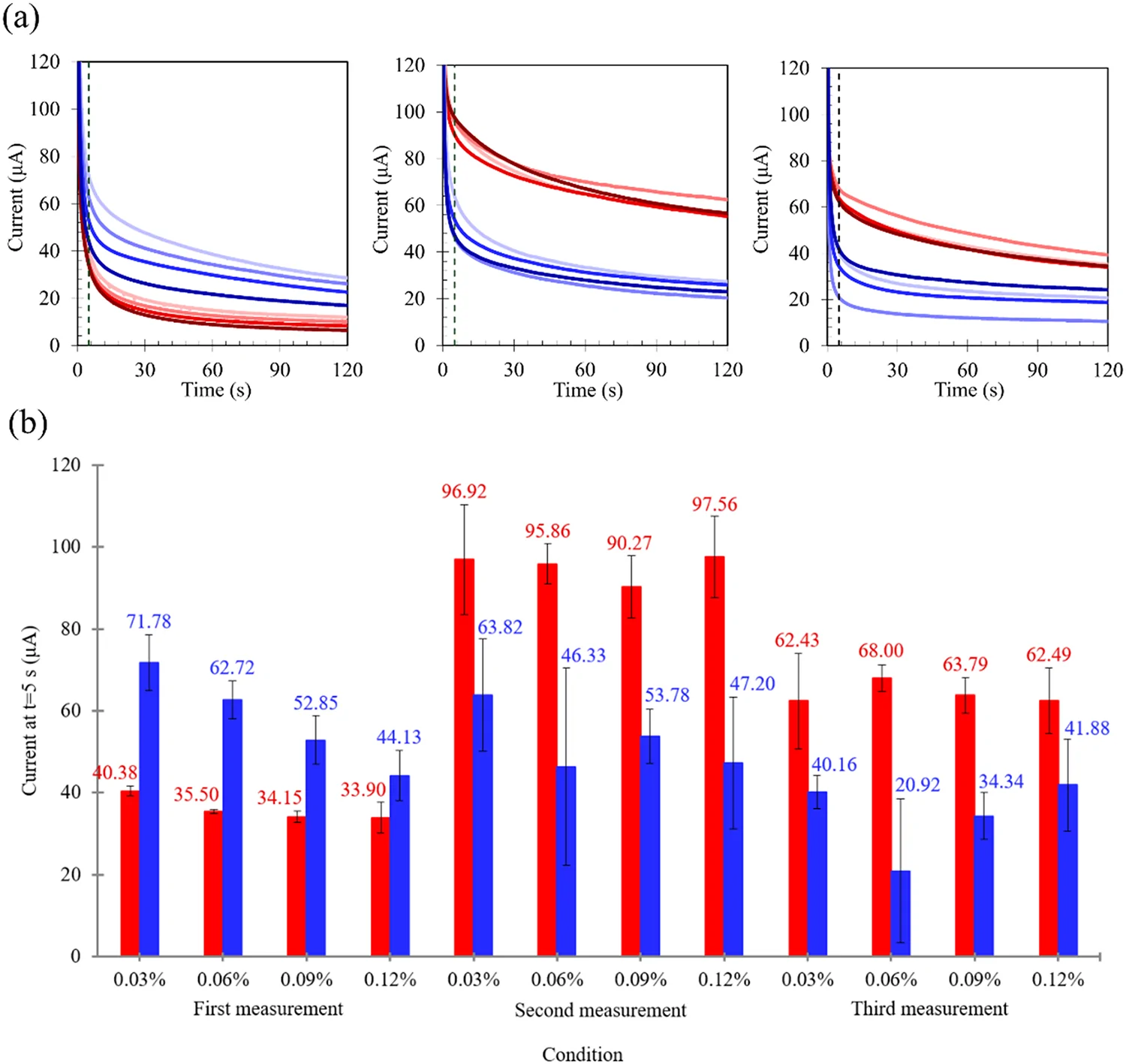
Electrochemical response of SPCE/Naf/CL biosensors functionalized with different cardiolipin concentrations (0.03%, 0.06%, 0.09%, and 0.12%) for the discrimination between positive and negative control materials. (a) Mean chronoamperometric profiles obtained from the first, second, and third consecutive measurements for positive and negative controls. Red chronoamperograms represent the positive control, whereas blue chronoamperograms represent the negative control, with progressively darker shades within each color corresponding to increasing cardiolipin concentrations. The dashed line indicates the 5 s time point at which the current values were extracted for subsequent analysis. (b) Electrical current values extracted at 5 s from each chronoamperometric measurement. Red bars represent the positive control, whereas blue bars represent the negative control. Error bars indicate the standard deviation obtained from four technical replicate electrodes per experimental condition (*n* = 4), and the values above each bar represent the corresponding mean current.

The temporal distribution of the electrical current, shown in Figure 4, revealed important differences in the discriminatory performance of the biosensor according to the electrochemical measurement.

During the first measurement (*i*_5,1_), positive control consistently exhibited lower current values than the negative control at all cardiolipin concentrations evaluated. This behavior, in which positive materials containing anticardiolipin antibodies produced lower electrical currents than negative materials, is consistent with the findings of Do Egito et al., who detected anticardiolipin antibodies through current blocking at the electrode/electrolyte interface and reported a progressive decrease in anodic current measured by cyclic voltammetry as syphilis antibody titers increased, reaching the lowest values for samples with a titer of 1:1024.^9^

Similarly, Shen et al. reported a decrease in oxidation–reduction peak currents resulting from biomolecule adsorption onto the electrode surface, attributing this effect to the formation of an interfacial layer that hinders both electron transfer and mass transport processes.^29^ Comparable results have also been reported for differential pulse voltammetry-based immunosensors, in which the decrease in peak current with increasing analyte concentration was associated with increased surface bulkiness following antigen–antibody complex formation, thereby slowing electron transfer and reducing the electrochemical response.^30^ These findings support the hypothesis that interactions between the antibody-containing positive control and the cardiolipin-functionalized interface may contribute to the lower current observed during the first measurement.

Moreover, regardless of cardiolipin concentration, positive control displayed similar current responses, with mean values ranging from 33.90 to 40.38 μA. In contrast, the current measured for the negative control progressively decreased as the cardiolipin concentration increased, resulting in greater separation between positive and negative control materials, particularly for the SPCE/Naf/CL3 (0.03% cardiolipin) and SPCE/Naf/CL6 (0.06% cardiolipin) electrodes. Notably, this concentration-dependent behavior was observed in the chronoamperometric response despite the absence of clearly distinguishable differences among cardiolipin concentrations during cyclic voltammetry and electrochemical impedance spectroscopy characterization. This apparent difference may be related to the distinct experimental conditions and electrochemical responses probed by each technique. For CV and EIS characterization, a ferro/ferricyanide redox probe was used, providing a defined electrochemical response for evaluating charge-transfer properties at the electrode interface. Under these conditions, the changes produced by the different cardiolipin concentrations may not have been sufficiently pronounced to be clearly distinguished.

In the chronoamperometric experiments, however, the control materials were applied directly to the functionalized electrodes without the addition of an exogenous redox probe. Considering the negative control, for which specific recognition by anticardiolipin antibodies is not expected, the progressive decrease in current with increasing cardiolipin concentration may be associated with changes in the physicochemical properties of the interface as the amount of immobilized cardiolipin increases. Greater surface coverage may result in the formation of a denser interfacial layer, potentially hindering species transport and/or charge transfer at the electrode–solution interface. Such an interfacial effect is consistent with previous electrochemical studies demonstrating that the amount of cardiolipin deposited on electrode surfaces can influence the compactness and permeability of the resulting lipid film.^31^ Nevertheless, because no defined redox probe was present during chronoamperometry, the recorded current cannot be unequivocally attributed to a specific faradaic process, and contributions from interfacial capacitive processes and electroactive components of the control matrix cannot be excluded under the present experimental conditions.

In the subsequent measurements (*i*_5,2_ and *i*_5,3_), an inversion of the electrochemical signal was observed, with positive control exhibiting higher currents than negative control. In contrast, negative control exhibited lower current values accompanied by greater intragroup variability, reducing discrimination between positive and negative materials, as illustrated by the larger error bars. No clear concentration-dependent pattern was observed for the negative control during this measurement.

The inversion observed after the first measurement should be interpreted considering the experimental conditions associated with the consecutive-measurement protocol. Throughout the experimental sequence, the control material remained in contact with the functionalized surface, allowing continued interaction at the electrode-control material interface, while the same electrode was also subjected to successive electrochemical polarizations. Thus, both continued interfacial interactions and repeated electrochemical stimulation may contribute to the changes observed across consecutive measurements, although their individual contributions cannot be independently determined under the present experimental design.

Interactions between the antibody-containing positive control and immobilized cardiolipin may progressively modify the physicochemical properties of the interface. In particular, antibody binding to immobilized cardiolipin may affect charge distribution and double-layer capacitance because the formation of a bulky and charged biomolecular layer can alter the electrical characteristics of the electrode/electrolyte interface.^32,33^ At the same time, successive electrochemical polarization may promote additional interfacial changes, including electrode conditioning, progressive Nafion hydration, surface occupation, nonspecific adsorption of matrix components, and rearrangement or partial desorption of interfacial species. Collectively, these processes may contribute to changes in both the magnitude and relative current responses of the positive and negative controls during consecutive measurements. Therefore, the inversion observed after *i*_5,1_ is more appropriately interpreted as the response of an evolving electrode-control material interface under continued contact and successive electrochemical stimulation, rather than being attributed exclusively to a single interfacial phenomenon.

A similar discrimination pattern was maintained during the third measurement (*i*_5,3_), with the positive control generally exhibiting higher currents than the negative control; however, a reduction in the magnitude of the measured current was observed for both control materials compared with the second measurement. Unlike *i*_5,2_, the third measurement revealed a clearer influence of cardiolipin concentration on the discrimination between the control materials. Electrodes functionalized with 0.03% and 0.12% cardiolipin showed poorer discrimination between positive and negative controls, as evidenced by the overlap of the corresponding current values and their associated error bars at specific time intervals.

At the lowest cardiolipin concentration (0.03%), the lower availability of immobilized antigen at the electrode surface may limit the extent of biomolecular interaction with the non-treponemal antibodies present in the positive control. Consequently, reduced immunocomplex formation could contribute to the lower discrimination observed between the positive and negative control materials. Conversely, at the highest cardiolipin concentration (0.12%), a greater abundance of immobilized antigen may affect the accessibility and organization of the interaction sites and, under conditions of antigen excess, potentially contribute to a response analogous to the postzone effect described in immunological systems.^34,35^ Although these concentration-dependent effects provide plausible explanations for the reduced discrimination observed at the lowest and highest cardiolipin concentrations, they should be interpreted together with the interfacial changes occurring during consecutive electrochemical measurements.

In addition to these concentration-dependent effects, repeated electrochemical measurements may progressively modify the electrode interface. Continuous adsorption of proteins and other components of the control matrix may promote surface occupation and biofouling, leading to changes in charge-transfer and mass-transport properties and reduced electrochemical signal stability during repeated measurements.^36^ Likewise, the ionic transport properties and conductivity of Nafion are strongly dependent on its hydration state and structural organization. Therefore, continued exposure to the aqueous control material, together with repeated electrochemical polarization, may progressively alter the characteristics of the Nafion layer and contribute to the variability observed during later measurements.^37^

Taken together, these results indicate that the electrochemical response during consecutive measurements likely reflects the combined contribution of ongoing interactions at the cardiolipin-functionalized surface and progressive interfacial changes associated with successive electrochemical stimulation. Although the present experimental design does not allow the individual contribution of these processes to be independently determined, the first measurement is obtained before the interface has been subjected to any previous electrochemical polarization. Accordingly, *i*_5,1_ provides the most direct assessment of the initial response of the control materials at the cardiolipin-functionalized interface. To further assess these differences and determine whether the response patterns observed across cardiolipin concentrations, control materials, and consecutive measurements were statistically supported, the current values extracted at 5 s were subsequently analyzed using a linear mixed-effects model.

The linear mixed-effects model revealed significant effects of control material (*F*(1, 24) = 54.399; *p* < 0.001) and measurement number (*F*(2, 24) = 79.046; *p* < 0.001). However, the effect of cardiolipin concentration did not reach statistical significance (*F*(3, 24) = 2.961; *p* = 0.052). Regarding the interactions between factors, no significant interaction was observed between cardiolipin concentration and control material (*F*(3, 24) = 2.103; *p* = 0.126). In contrast, significant interactions were observed between cardiolipin concentration and measurement number (*F*(6, 24) = 3.937; *p* = 0.007) and between control material and measurement number (*F*(2, 24) = 226.917; *p* < 0.001). Furthermore, the three-way interaction among cardiolipin concentration, control material, and measurement number was significant (*F*(6, 24) = 4.005; *p* = 0.006), indicating that the difference between the positive and negative control materials across consecutive measurements varied according to cardiolipin concentration.

Given the significant three-way interaction, post hoc comparisons were performed between the positive and negative control materials within each combination of cardiolipin concentration and measurement number, with Holm correction for multiple comparisons. The mean differences between control materials, their corresponding 95% confidence intervals (CI), and Holm-adjusted *p*-values are presented in Table S1. During *i*_5,1_, the positive control material exhibited significantly lower currents than the negative control material at all cardiolipin concentrations evaluated. In the subsequent measurements (*i*_5,2_ and *i*_5,3_), the direction of the response was reversed, with significantly higher currents observed for the positive control material compared with the negative control material. All comparisons between the positive and negative control materials remained statistically significant after Holm correction.

Assessment of the model assumptions indicated an overall adequate behavior of the residuals. Visual inspection of the Q–Q plot (Figure S2a) showed an approximately normal distribution of the residuals, with most points lying close to the reference line, although some deviations were observed in the tails. Additionally, the residuals-versus-fitted-values plot showed that the residuals were distributed around zero, with no evident systematic pattern or pronounced heteroscedasticity (Figure S2b). Therefore, the residual analysis did not indicate substantial violations of the model assumptions that would compromise the interpretation of the results.

Overall, the SPCE/Naf/CL6 biosensor during the first chronoamperometric measurement exhibited the most favorable analytical performance, combining low intragroup variability among replicate electrodes for both positive and negative controls with a pronounced separation between the two control groups. Based on this combination of response consistency and discriminatory capacity, the SPCE/Naf/CL6 condition and the first chronoamperometric measurement were selected for subsequent experiments.

### 3.3 Elucidating the Mechanism of the Electrochemical Reaction through Microscopic Evaluation

Figure 5 presents the microscopic evaluation of the interaction between the antigen–antibody complex using a positive and negative control material and the commercial antigen suspension present in the VDRL kit (Figure 5a,b), as well as using the positive control material combined with different concentrations of cardiolipin antigen solution (Figure 5c–f).

**Figure 5:**
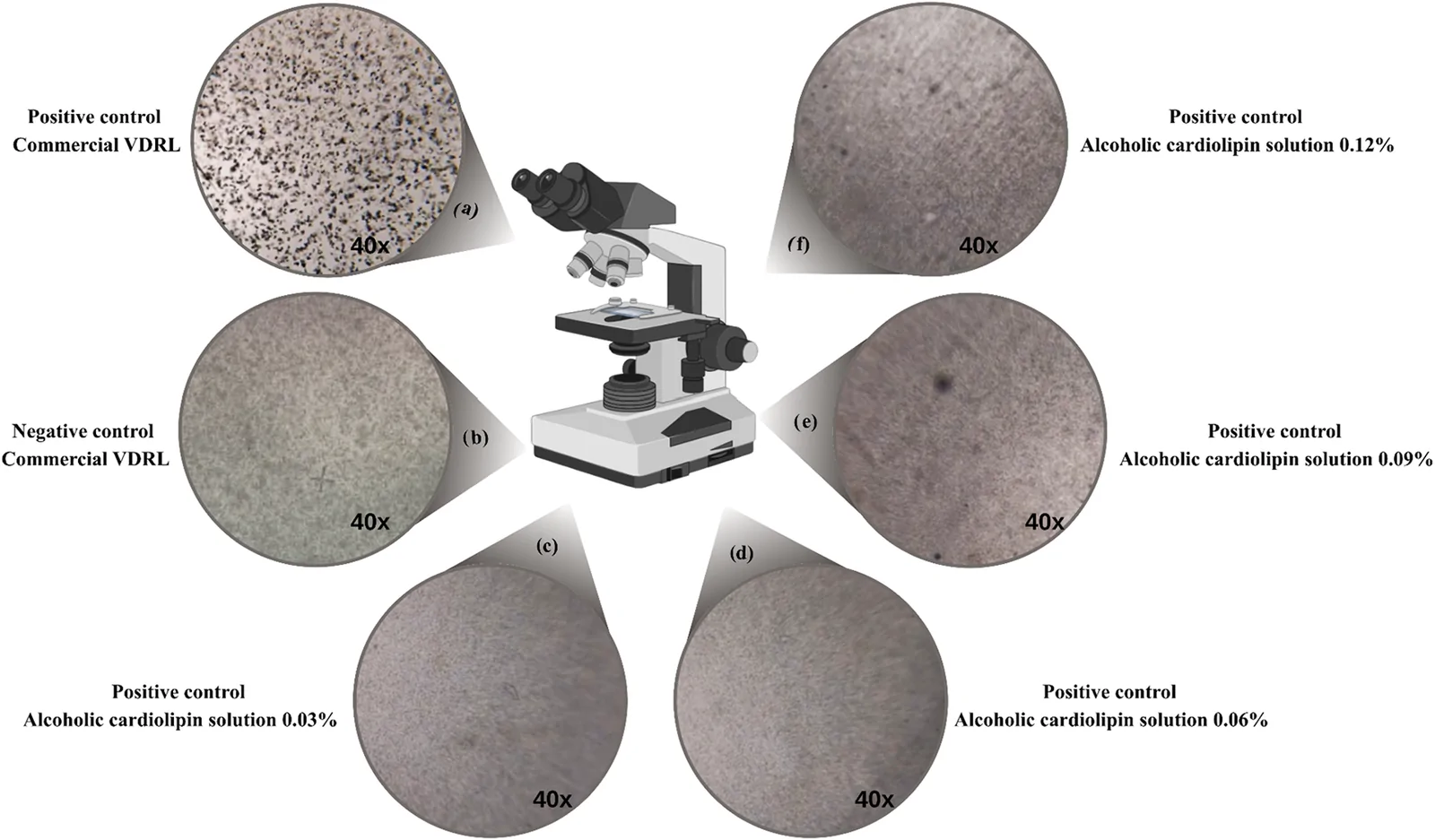
Microscopic analysis of the interaction between antigen and antibody: (a) commercial VDRL antigen suspension with positive control; (b) commercial VDRL antigen suspension with negative control; (c–f) cardiolipin solutions at concentrations of 0.03%, 0.06%, 0.09%, and 0.12%, respectively, with positive control. Images were obtained using a 40× total magnification.

As observed in Figure 5a, the use of the commercial VDRL antigen suspension in the presence of the positive control results in the formation of well-defined aggregates, characteristic of a flocculation reaction. In contrast, in the presence of the negative control (Figure 5b), no aggregate formation was observed, and the suspension maintained a homogeneous appearance. This result is consistent with the absence of detectable anticardiolipin antibodies in the negative control material and, consequently, with the absence of the antigen–antibody interaction required to promote flocculation.

The formation of aggregates observed in the positive control is not only dependent on the interaction between anticardiolipin antibodies and cardiolipin, but is also influenced by the presence of lecithin and cholesterol in the VDRL antigen formulation. Although lecithin and cholesterol are not directly involved in immunological recognition between cardiolipin and anticardiolipin antibodies, they play key roles in promoting the structural organization of the lipid system required for the formation of microscopically detectable flocculation. Lecithin acts as a dispersing agent and promotes the formation of liposomal structures, being essential for obtaining clear results in flocculation tests, in addition to significantly enhancing cardiolipin reactivity. Cholesterol, on the other hand, acts as an auxiliary lipid, contributing to the organization and stability of these structures, although its effect on reactivity may vary depending on the system composition.^38^ The literature also indicates that, in the absence of cholesterol and lecithin, this visualization is impaired, although cardiolipin is the antigen responsible for specific binding with anticardiolipin antibodies.^39^

For this reason, under conditions in which only cardiolipin in alcoholic solution was used (Figure 5c–f), without the presence of cholesterol and lecithin, no flocculation was observed, even in the presence of antibodies in the positive control material. This result is consistent with previous reports showing that isolated cardiolipin does not reproduce the characteristic flocculation observed with the complete VDRL antigen complex, although it retains the ability to interact with cardiolipin-reactive antibodies.^40^ Accordingly, the absence of flocculation suggests that the electrochemical response observed in the present system does not depend on the macroscopic lipid aggregation phenomenon characteristic of the conventional VDRL assay, but may instead reflect interfacial interactions occurring between the antibody-containing control material and the cardiolipin-functionalized electrode surface.

Importantly, isolated cardiolipin and the cardiolipin-cholesterol-lecithin antigen complex used in conventional VDRL testing should not be considered immunologically equivalent. Koike et al. demonstrated that antibody populations may exhibit different recognition patterns toward isolated cardiolipin and the complete VDRL antigen, indicating that removal of cholesterol and lecithin may influence antigen recognition.^41^ In the present preliminary proof-of-concept study, isolated cardiolipin was therefore used as an immobilized recognition element to investigate whether antibody-containing commercial VDRL control materials could produce a distinguishable electrochemical response, rather than with the assumption that isolated cardiolipin fully reproduces the immunological behavior of the conventional VDRL antigen complex.

This finding is particularly relevant in the context of this work, as it reinforces that the electrochemical response in the developed biosensor is directly related to the molecular recognition event, and not to secondary flocculation mechanisms characteristic of conventional non-treponemal tests, without requiring incubation times. The first measurement (*i*_5,1_) previously presented in Figure 4 showed the greatest discrimination between positive and negative controls, suggesting that the initial electrochemical response is more sensitive to interactions occurring at the cardiolipin-functionalized interface. In the second (*i*_5,2_) and third (*i*_5,3_) consecutive measurements, the reduced discrimination may be associated with progressive changes at the electrode–sample interface, including surface occupation by adsorbed components, interfacial rearrangement, and nonspecific adsorption. These processes may modify charge transfer during repeated measurements, thereby affecting the magnitude of the electrochemical response and reducing the distinction between positive and negative controls.^36,42^

Although the obtained results support the feasibility of this electrochemical sensing strategy, further studies using well-characterized clinical samples will be required to determine whether the platform can distinguish non-treponemal antibody reactivity associated with syphilis from cardiolipin-reactive antibodies occurring in other clinical conditions.

### 3.4 Effect of Non-treponemal Antibodies Dilution on the Electrochemical Response

The two electrode batches used in this study had the same composition and showed no significant differences in their baseline electrochemical characterization by voltammetry, as presented in the Supporting Information (Figure S3). These results indicate comparable electrochemical behavior of the SPCEs prior to immobilization. Figure 6 presents the electrochemical responses obtained for the positive and negative control materials after cardiolipin immobilization using electrodes from both batches.

**Figure 6:**
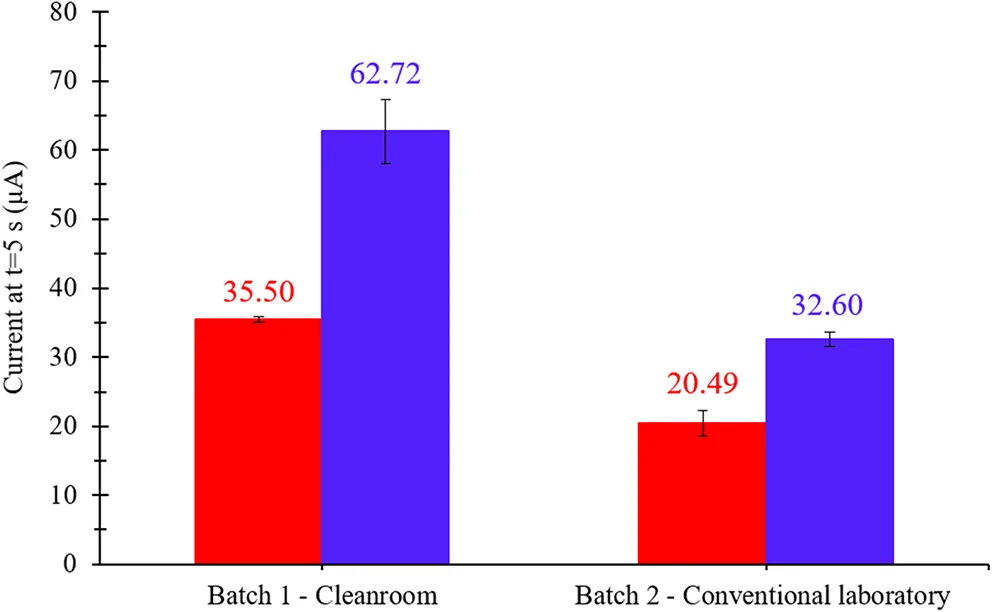
Comparison of the electrochemical response of SPCE/Naf/CL6 biosensors from two different electrode batches evaluated under the same experimental conditions for the discrimination between positive and negative control materials. Electrical current values were extracted at 5 s from the chronoamperometric measurements. Red bars represent the positive control, whereas blue bars represent the negative control. Error bars indicate the standard deviation obtained from four technical replicate electrodes per experimental condition (*n* = 4), and the values above each bar represent the corresponding mean current.

As shown in Figure 6, a reduction in the magnitude of the electrical current was observed for the second batch compared with the first. Despite this difference in the absolute signal magnitude, both experimental sets consistently retained the ability to discriminate between the positive and negative control materials.

Considering that no significant differences were identified between the unmodified electrodes from the two batches, the variation observed after immobilization may be related to the environmental conditions employed during biosensor preparation and evaluation. Experiments performed with the first batch, including the cardiolipin immobilization step, were conducted in a Class 100,000 (ISO 8) cleanroom under controlled temperature (20 ± 5 °C) and relative humidity (50 ± 5%) conditions. In contrast, the immobilization and experiments performed with the second batch were conducted in a conventional laboratory without specific control of these environmental parameters, with the aim of evaluating the behavior of the platform under conditions more closely resembling a real-world testing setting. Therefore, differences in temperature and, particularly, humidity during immobilization and/or electrochemical measurements may have contributed to the variation in current magnitude observed between the experimental sets.

Future studies should systematically investigate the influence of temperature and humidity on the functionalization, stability, and electrochemical response of the platform, as well as establish its stability during storage. For this purpose, electrodes stored in packaging designed to protect against light and moisture, including the use of desiccant materials, will be evaluated over predefined storage periods.^22^ These studies will allow the stability of the functionalized layer to be determined, appropriate storage and packaging conditions to be established, and the shelf life of the biosensors to be assessed prior to their application under real-world conditions.

Based on the optimized condition established in the previous section, serial dilutions of the positive control material (1:2, 1:4, 1:8, and 1:16) were evaluated to investigate the biosensor’s ability to detect gradual variations in the electrochemical response resulting from control material dilution, considering that the 1:2 dilution represents a higher relative proportion of the antibody-containing positive control material than the 1:16 dilution. Figure 7 presents the electrochemical responses obtained using SPCE/Naf/CL6 electrodes across the different dilution factors of the positive control material.

**Figure 7:**
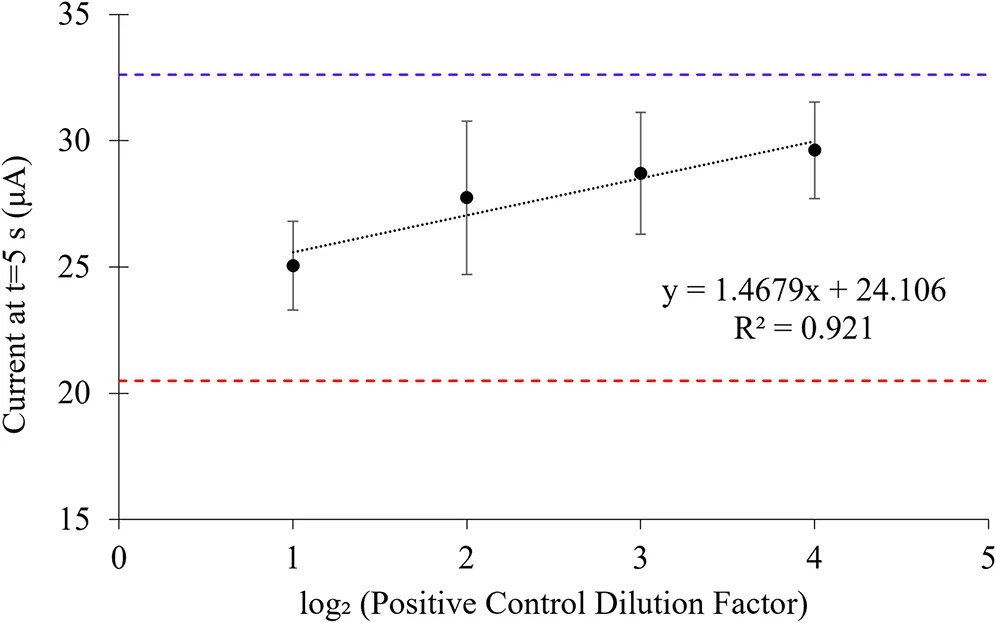
Evaluation of the electrochemical response of the SPCE/Naf/CL6 biosensor toward serial dilutions of the positive control compared to the negative control. The points represent the average electrical current measured over 5 s during the first chronoamperometric analysis for the 1:2, 1:4, 1:8, and 1:16 dilutions, expressed as a dilution factor on a log_2_ scale. Error bars correspond to the standard deviation of experimental quadruplicates. The dashed horizontal lines indicate the mean values obtained for the positive (red) and negative (blue) control materials. The discrimination thresholds were established based on the electrical current responses obtained for the positive and negative control materials using electrodes from the second batch. The dotted line represents the linear fit of the experimental data, with the equation and *R* ^2^ shown in the figure.

As shown in Figure 7, a progressive change in the electrochemical response was observed as the positive control material underwent successive dilutions. Lower dilutions, corresponding to a higher relative proportion of the antibody-containing positive control material, exhibited lower current values, whereas higher dilutions resulted in progressively higher currents. This behavior may be associated with interactions between immobilized cardiolipin and the antibodies present in the positive control material, leading to changes at the electrode–control material interface. At lower dilutions, the higher relative proportion of antibody-containing positive control material may favor greater interaction with the cardiolipin-functionalized surface, potentially increasing interfacial blocking and contributing to the lower current values observed. In contrast, progressive dilution of the positive control material may reduce the extent of these interfacial interactions, resulting in progressively higher current values. This interpretation is consistent with the behavior reported for electrochemical biosensors in which biomolecular recognition events induce changes at the electrode interface and consequently affect the measured electrochemical response.^9^

In addition, the relationship between the measured electrical current and the material dilution factor showed high linearity, with an *R* ^2^ of 0.921. This result demonstrates a strong relationship between the progressive dilution of the positive control material and the electrochemical response of the biosensor, indicating that the platform is responsive to changes in the relative amount of antibody-containing control material.^22^

The observed linear relationship between the electrochemical response and the dilution of the positive control material provides further evidence of the feasibility of the proposed platform. In addition to distinguishing between positive and negative control materials, the electrochemical response varied systematically as the positive control was progressively diluted in the negative control. Considering that non-treponemal tests conventionally rely on serial dilution to evaluate serological reactivity, these findings demonstrate that the developed platform is responsive to changes in the relative amount of antibody-containing control material. Although the present proof-of-concept was not designed to establish a formal limit of discrimination or determine antibody titers, the results support further investigation of the platform using specimens with defined serological titers and appropriate analytical validation.

### 3.5 Effect of Cardiolipin on Electrochemical Response and Interference Testing

To investigate the contribution of immobilized cardiolipin to the electrochemical response, positive and negative control materials were evaluated using SPCE/Naf/CL6 and SPCE/Naf electrodes. Figure 8 presents the electrochemical responses obtained for both control materials in the presence and absence of immobilized cardiolipin.

**Figure 8:**
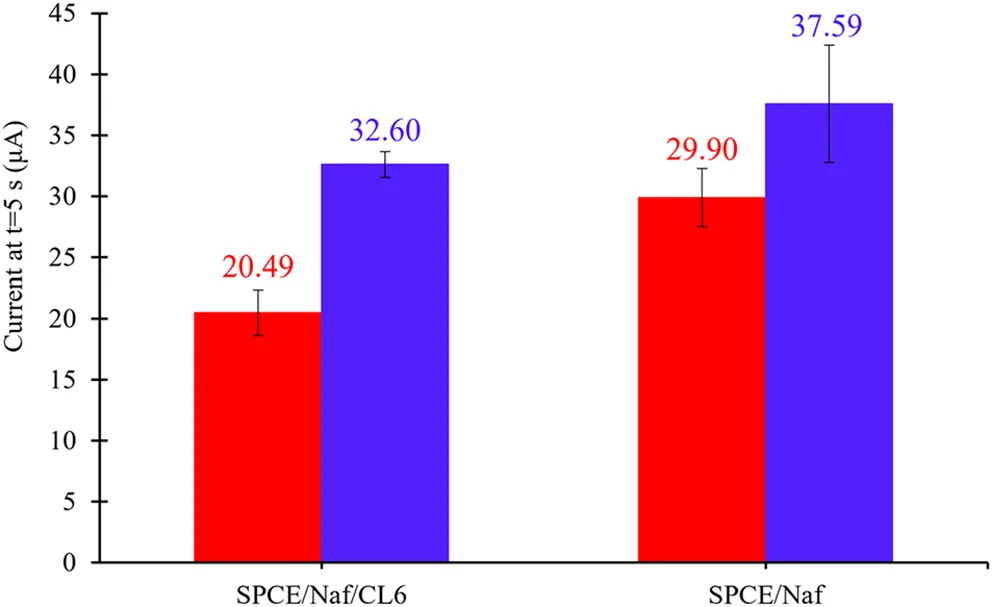
Effect of immobilized cardiolipin on the electrochemical response to positive and negative controls. Electrical current values extracted at 5 s from chronoamperometric measurements performed using electrodes modified with Nafion and 0.06% cardiolipin (SPCE/Naf/CL6) and electrodes modified with Nafion only (SPCE/Naf). Red bars represent the positive control, whereas blue bars represent the negative control. Error bars indicate the standard deviation obtained from four technical replicate electrodes per experimental condition (*n* = 4), and the values above each bar represent the corresponding mean current.

As shown in Figure 8, when the control materials were evaluated using electrodes without immobilized cardiolipin (SPCE/Naf), the same overall response pattern observed for SPCE/Naf/CL6 was maintained, with the negative control exhibiting a higher mean current than the positive control. However, the separation between the positive and negative controls was more pronounced for electrodes containing immobilized cardiolipin (SPCE/Naf/CL6). For the positive control, SPCE/Naf/CL6 exhibited a lower mean current at 5 s (20.49 ± 1.87 μA) than SPCE/Naf (29.90 ± 2.38 μA). This decrease in current following cardiolipin immobilization may be associated with changes at the electrode-control material interface resulting from interactions between immobilized cardiolipin and the non-treponemal antibodies present in the positive control.

Similarly, the negative control exhibited a lower mean current at 5 s for SPCE/Naf/CL6 (32.60 ± 1.07 μA) than for SPCE/Naf (37.59 ± 4.81 μA). Although the negative control exhibited a higher mean current than the positive control under both conditions, greater within-group variability was observed for SPCE/Naf, resulting in less distinct separation between the control responses. In contrast, SPCE/Naf/CL6 showed greater numerical separation between the positive and negative controls, together with lower within-group variability among the technical replicate electrodes.

Factorial ANOVA (complete results are presented in Table S2) revealed significant main effects of control material (*F*(1, 12) = 46.974, *p* < 0.001) and electrode immobilization (*F*(1, 12) = 24.790, *p* < 0.001) on the current recorded at 5 s. The significant effect of control material indicates that the system responds differently to positive and negative control materials, while the significant effect of electrode immobilization demonstrates that the electrode functionalization strategy effectively influences the analytical response.

Simple-effect analyses showed that the positive and negative control materials differed significantly for both SPCE/Naf/CL6 (*F*(1, 6) = 126.143, *p* < 0.001) and SPCE/Naf (*F*(1, 6) = 8.235, *p* = 0.028). When the effect of immobilization was evaluated within each control material, SPCE/Naf/CL6 exhibited a significantly lower current than SPCE/Naf for the positive control (*F*(1, 6) = 38.635, *p* = 0.001), whereas the difference between immobilization did not reach statistical significance for the negative control (*F*(1, 6) = 4.099, *p* = 0.089). The mean differences and their corresponding 95% CIs are presented in Table S3.

Taken together, these findings demonstrate that cardiolipin immobilization significantly influenced the electrochemical response of the system. Notably, the presence of immobilized cardiolipin was associated with a significant decrease in the current recorded for the positive control material, whereas the corresponding change observed for the negative control did not reach statistical significance. In addition, SPCE/Naf/CL6 exhibited a greater numerical separation between the positive and negative control materials and lower within-group variability compared with SPCE/Naf. These findings support the contribution of immobilized cardiolipin to the electrochemical response and are consistent with the involvement of cardiolipin-antibody interactions at the electrode-control material interface.

Although these results support the contribution of immobilized cardiolipin to the discrimination between the commercial positive and negative controls, these materials have a simplified composition and do not reproduce the complexity of biological matrices. Therefore, to further investigate whether the observed electrochemical response could be influenced by electroactive compounds other than the target antibodies, additional experiments were performed in the presence of potential interfering species, including glucose, which may undergo electrochemical oxidation within the potential range employed in the chronoamperometric measurements. The results obtained in the absence of interferents were compared with those obtained in the presence of glucose at a physiological concentration (99 mg dL^–1^) and a pathological concentration (200 mg dL^–1^), as presented in Figure 9.

**Figure 9:**
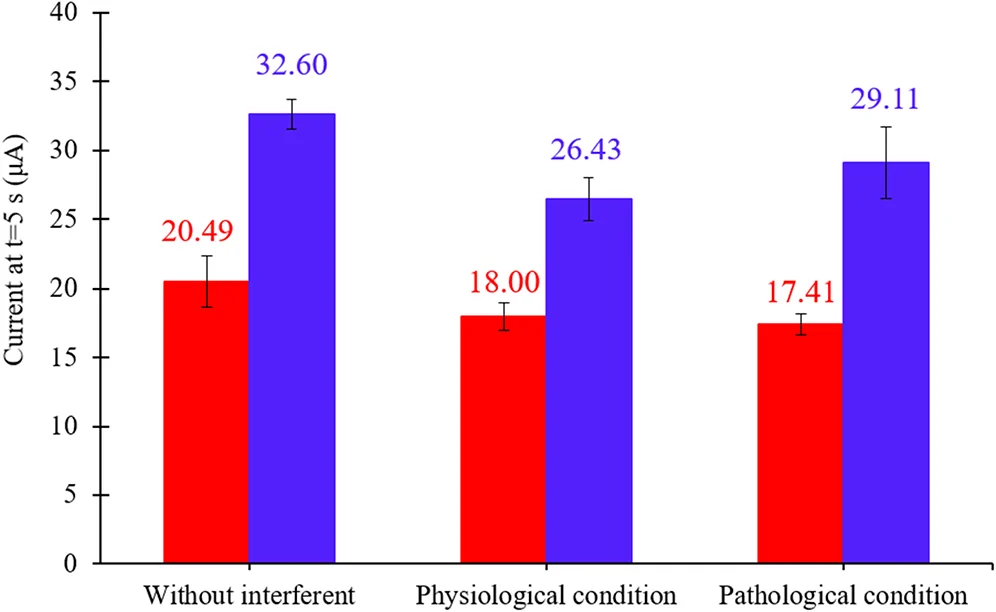
Effect of glucose interference on the electrochemical response of positive and negative control materials using SPCE/Naf/CL6 biosensor. Electrical current values were recorded at 5 s in the absence of interference and in the presence of glucose at concentrations of 99 mg dL^–1^ and 200 mg dL^–1^. Red bars represent the positive control material, and blue bars represent the negative control material. Data are presented as mean ± standard deviation (*n* = 4 technical replicate electrodes per condition).

In all conditions presented in Figure 9, the electrochemical response pattern between the control materials was maintained, with the current recorded at 5 s for the positive control remaining lower than that observed for the negative control. In the absence of glucose, mean currents of 20.49 ± 1.87 μA and 32.60 ± 1.07 μA were obtained for the positive and negative controls, respectively. In the presence of glucose at 99 mg dL^–1^, corresponding to the upper limit of the normal fasting glucose range, the mean currents were 18.00 ± 1.00 μA for the positive control and 26.43 ± 1.56 μA for the negative control. Similarly, in the presence of 200 mg dL^–1^ glucose, representing a markedly elevated glucose concentration, mean currents of 17.41 ± 0.73 μA and 29.11 ± 2.60 μA were observed for the positive and negative controls, respectively.

Considering the mean values and their respective standard deviations, the presence of glucose at both concentrations evaluated did not prevent differentiation between the positive and negative control materials, and the same response pattern observed in the absence of the interferent was maintained. These results indicate that, under the experimental conditions evaluated, glucose, including at a concentration of 200 mg dL^–1^, did not compromise the ability of the platform to discriminate between the control materials This finding is relevant because the contribution of electroactive interferents to amperometric responses depends on the electrode material, surface functionalization, and applied potential, and such species may contribute to the measured current when their electrochemical reactions occur within the operating potential window.^43,44^

Factorial ANOVA (complete results are presented in Table S4) revealed a statistically significant difference between positive and negative control materials across all conditions with and without interferents (*F*(1, 18) = 270.579, *p* < 0.001). Additionally, a statistically significant difference was observed regarding the presence or absence of glucose in both control materials (*F*(2, 18) = 16.001, *p* < 0.001).

Pairwise comparisons further showed that the positive and negative control materials remained significantly different in the absence of interferent and in the presence of glucose at both 99 and 200 mg dL^–1^, with Bonferroni-adjusted *p*-values <0.001 for all comparisons. The mean differences between the control materials and their corresponding 95% CIs are presented in Table S5.

Pairwise comparisons among glucose conditions within each control material (complete results are presented in Table S6) showed that, for the positive control, the current obtained under the pathological glucose condition (200 mg dL^–1^) differed significantly from that obtained in the absence of interferent (Bonferroni-adjusted *p* = 0.041), whereas no significant differences were observed for the other pairwise comparisons. This finding suggests that, in the positive control, glucose at the pathological concentration may influence the electrochemical response, while no statistically significant effect was detected at the physiological concentration (99 mg dL^–1^).

For the negative control, the current measured in the absence of interferent differed significantly from that observed in the presence of both physiological (99 mg dL^–1^; Bonferroni-adjusted *p* < 0.001) and pathological (200 mg dL^–1^; Bonferroni-adjusted *p* = 0.019) glucose concentrations. In contrast, no significant difference was observed between the physiological and pathological glucose conditions (*p* = 0.088). Therefore, for the negative control, the observed effect cannot be attributed exclusively to the pathological glucose concentration, suggesting that the presence of glucose itself may influence the electrochemical response.

Overall, the positive control appeared less susceptible to glucose-related changes than the negative control, particularly at the physiological glucose concentration. One possible explanation may be the specific antigen–antibody interaction occurring in the positive samples, in which cardiolipin-containing antigenic components promote the recognition of non-treponemal antibodies. This specific binding may contribute to a more robust analytical response and potentially reduce the relative impact of matrix-related electrochemical effects. Nevertheless, since no significant difference was observed between the physiological and pathological glucose concentrations for either control material, the results do not support a clear concentration-dependent interference effect of glucose. Therefore, although glucose influenced the magnitude of the measured current, it did not prevent electrochemical discrimination between the positive and negative control materials under the experimental conditions evaluated.

Although the glucose interference experiment provides an initial assessment of the influence of an electroactive species on the measured response, it does not address potential immunological cross-reactivity. This represents an important limitation of the present study, particularly because anticardiolipin and other antiphospholipid antibodies may contribute to biological false-positive reactions in conventional non-treponemal tests. Therefore, future studies should evaluate the proposed platform using appropriately characterized clinical specimens, including specimens containing antiphospholipid antibodies and VDRL-negative specimens from healthy pregnant women, to determine its clinical specificity and suitability for the intended target population.

## 4 Conclusion

Gestational and congenital syphilis remain significant public health challenges, particularly because of persistent vertical transmission and the consequences associated with delayed diagnosis and inadequate treatment. Non-treponemal testing plays an important role in syphilis screening and follow-up, while serological titers provide clinically relevant information for monitoring treatment response. In this context, the development of alternative analytical platforms capable of providing rapid and objective assessment of non-treponemal antibody reactivity may contribute to future strategies for decentralized testing.

In this preliminary proof-of-concept study, an amperometric biosensor based on isolated cardiolipin as the sole biorecognition element immobilized on carbon/graphene screen-printed electrodes was developed and evaluated using commercial positive and negative VDRL control materials. The proposed platform successfully discriminated between the positive and negative controls, while progressive dilution of the positive control material produced a systematic variation in the electrochemical response, demonstrating the responsiveness of the platform to changes in the relative proportion of antibody-containing control material.

The additional experiments performed in this study further supported the feasibility of the proposed sensing strategy. Two independent electrode batches were evaluated, and both consistently maintained discrimination between the positive and negative control materials, despite differences in absolute current magnitude between experimental sets. Comparison of electrodes with and without immobilized cardiolipin demonstrated that cardiolipin functionalization significantly influenced the electrochemical response, particularly for the positive control material, supporting the contribution of the biorecognition element to the observed response. Preliminary interference testing showed that glucose influenced the magnitude of the measured current; nevertheless, significant discrimination between positive and negative control materials was maintained at both physiological (99 mg dL^–1^) and pathological (200 mg dL^–1^) glucose concentrations under the experimental conditions evaluated.

Further studies should focus on comprehensive reproducibility, storage stability, and clinical validation (using well-characterized clinical samples) to assess its potential for semiquantitative evaluation of non-treponemal antibody reactivity by systematically correlating the electrochemical response with defined serological titers. These advances may support the translation of the proposed platform toward decentralized syphilis testing and monitoring.

## Supplementary Material



## Notes

Additional electrochemical characterizations and statistical analyses supporting the results presented in the manuscript, the electrochemical characterization includes cyclic voltammetry of unmodified SPCE electrodes, Nafion-modified electrodes (SPCE/Naf), and electrodes modified with Nafion and different concentrations of immobilized cardiolipin (SPCE/Naf/CL), as well as the characterization and comparison of unmodified SPCE electrodes from two different batches, the post hoc comparisons between positive and negative control materials according to cardiolipin concentration and chronoamperometric measurements; diagnostic plots of residuals from the linear mixed-effects model; two-way factorial ANOVA evaluating the effects of control material and electrode immobilization on the electrochemical response; simple-effect comparisons between control materials and electrode immobilization conditions; two-way factorial ANOVA evaluating the effects of control material and glucose conditions; and pairwise comparisons between control materials within each glucose condition and among glucose conditions within each control material (PDF)

## Abbreviations

BIOSENS: Biosens Development and Biosensors Industry LTDA
UNISINOS: University of Vale do Rio dos Sinos
EPFL: Federal Institute of Technology Lausanne
VDRL: Venereal Disease Research Laboratory
T. pallidum: *Treponema pallidum*
TTs: Treponemal tests
NTTs: non-treponemal tests
RPR: Rapid Plasma Reagin
CV: Cyclic voltammetry
EIS: Electrochemical impedance spectroscopy
SPCE/Naf/CL: Sensors containing Nafion and cardiolipin
Ag/AgCl: Silver/silver chloride
SPCE: Unmodified sensors
SPCE/Naf: Sensors modified with only the Nafion layer
K_4_[Fe­(CN)_6_]: Potassium ferrocyanide
KCl: Potassium chloride
*i*_pc_: Cathodic peak current
*i*_pa_: Anodic peak current
Δ*E*: peak-to-peak potential separation
*E*_pa_: anodic peak potential
*E*_pc_: cathodic peak potential
|*i*_pa_/*i*_pc_|: absolute value of the peak current ratio
[Fe­(CN)_6_]^3–^/^4–^: Potassium ferri/ferrocyanide
OCP: Open-circuit potential
Rct: Charge-ransfer resistance
*i*_5,1_: First measurement
*i*_5,2_: Second measurement
*i*_5,3_: Third measurement
UN: Unstructured covariance structure
REML: Restricted maximum likelihood
Q–Q: Quantile–quantile
*R* ^2^: Coefficient of determination
CI: Confidence intervals
GLM: General Linear Model
