# Supplementary material for: Cardiolipin-Based Electrochemical Biosensor for Non-treponemal Antibody Detection Using Screen-Printed Electrodes: A Proof-of-Concept Study
Source: ACS Omega. 2026 Oct 27;11(44):68754–70. doi: 10.1021/acsomega.6c07930 (PMC13756735; doi:10.1021/acsomega.6c07930)
Supplement: Supplementary file 1 [file ao6c07930_si_001.pdf]

SUPPORTING INFORMATION

Cardiolipin-Based Electrochemical Biosensor for Non-treponemal Antibody Detection Using Screen-Printed Electrodes: A Proof-of-Concept Study

Duane da Silva Moraes[1,2], Bruna Ferri Serafini[2,3], Felipe Longaray Kadel[2,4], Gabriela Victória de Mello Jantzch[2,5], Milleny Germann Souza[2,6], Willyan Hasenkamp Carreira[2,7], Antonio Shigueaki Takimi[1], and Julia Konzen Moreira[2,6]*

1 Postgraduate Program in Mining, Metallurgical and Materials Engineering, Federal University of Rio Grande do Sul (UFRGS), Porto Alegre, RS, 91501-970, Brazil;

2 Biosens Development and Industry of Biosensors LTDA (BIOSENS), São Leopoldo, RS, 93022-715, Brazil;

3 Postgraduate Program in Collective Health, University of Vale do Rio dos Sinos (UNISINOS), São Leopoldo, RS, 93022-000, Brazil.

4 Undergraduate Program of Materials Engineering, Federal University of Rio Grande do Sul (UFRGS), Porto Alegre, RS, 91501-970, Brazil;

5 Postgraduate Program in Environmental Quality, Feevale University, Novo Hamburgo, RS, 93525-075, Brazil;

6 Postgraduate Program in Chemistry, Federal University of Rio Grande do Sul (UFRGS), Porto Alegre, RS, 91540-000, Brazil;

7 Postgraduate Program in Bioengineering and Biotechnology, Swiss Federal Institute of Technology Lausanne (EPFL), Lausanne, 1015, Switzerland.

* Corresponding author: duanedasilvamoraes@gmail.com

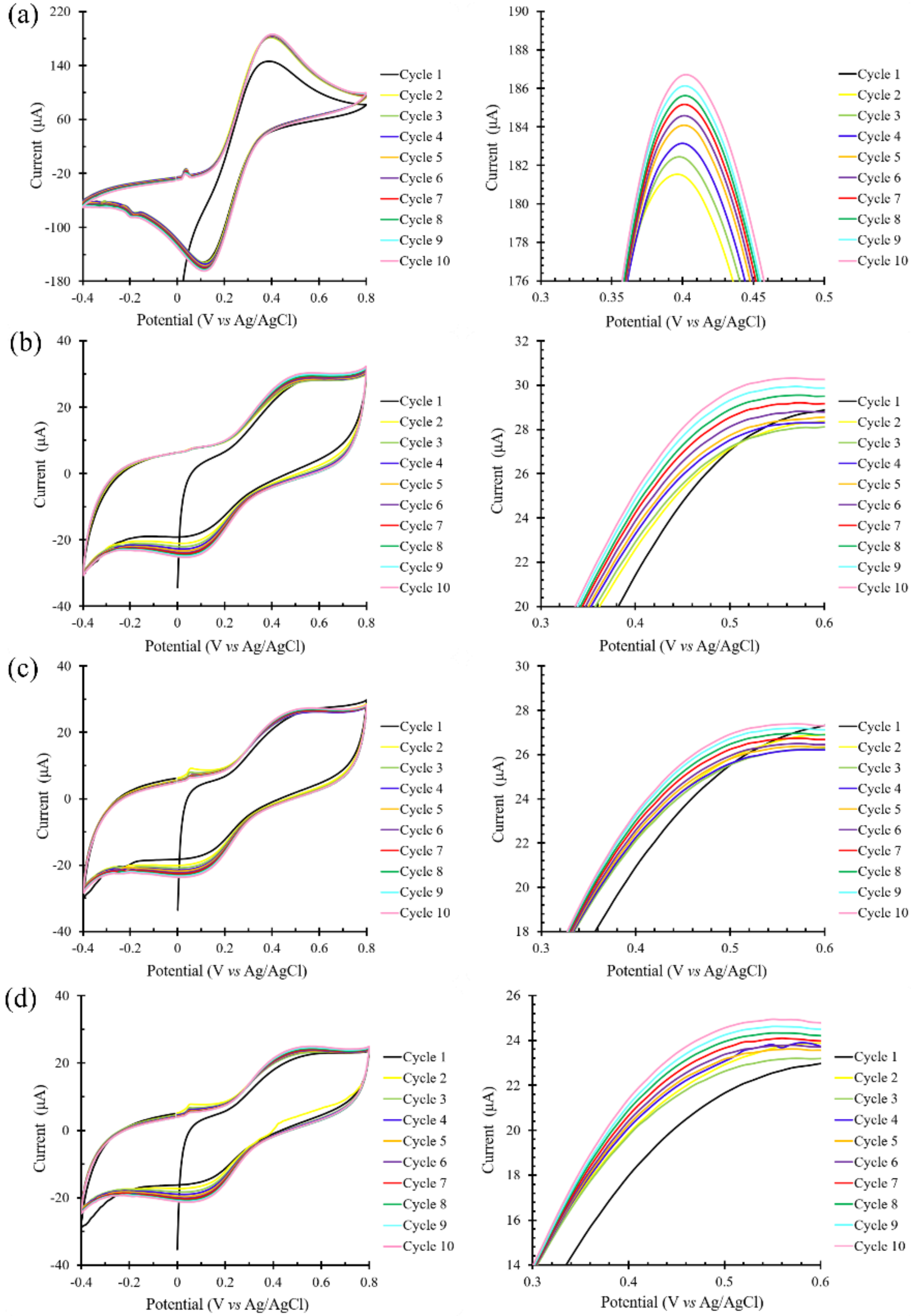
(a)
(b)
(c)
(d)
Current (μA)
Potential (V vs Ag/AgCl)
Cycle 1
Cycle 2
Cycle 3
Cycle 4
Cycle 5
Cycle 6
Cycle 7
Cycle 8
Cycle 9
Cycle 10
220
140
60
-20
-100
-180
190
188
186
184
182
180
178
176
40
20
0
-20
-40
32
30
28
26
24
22
20
18
16
14
-0.4
-0.2
0
0.2
0.4
0.6
0.8
0.3
0.35
0.4
0.45
0.5
0.6

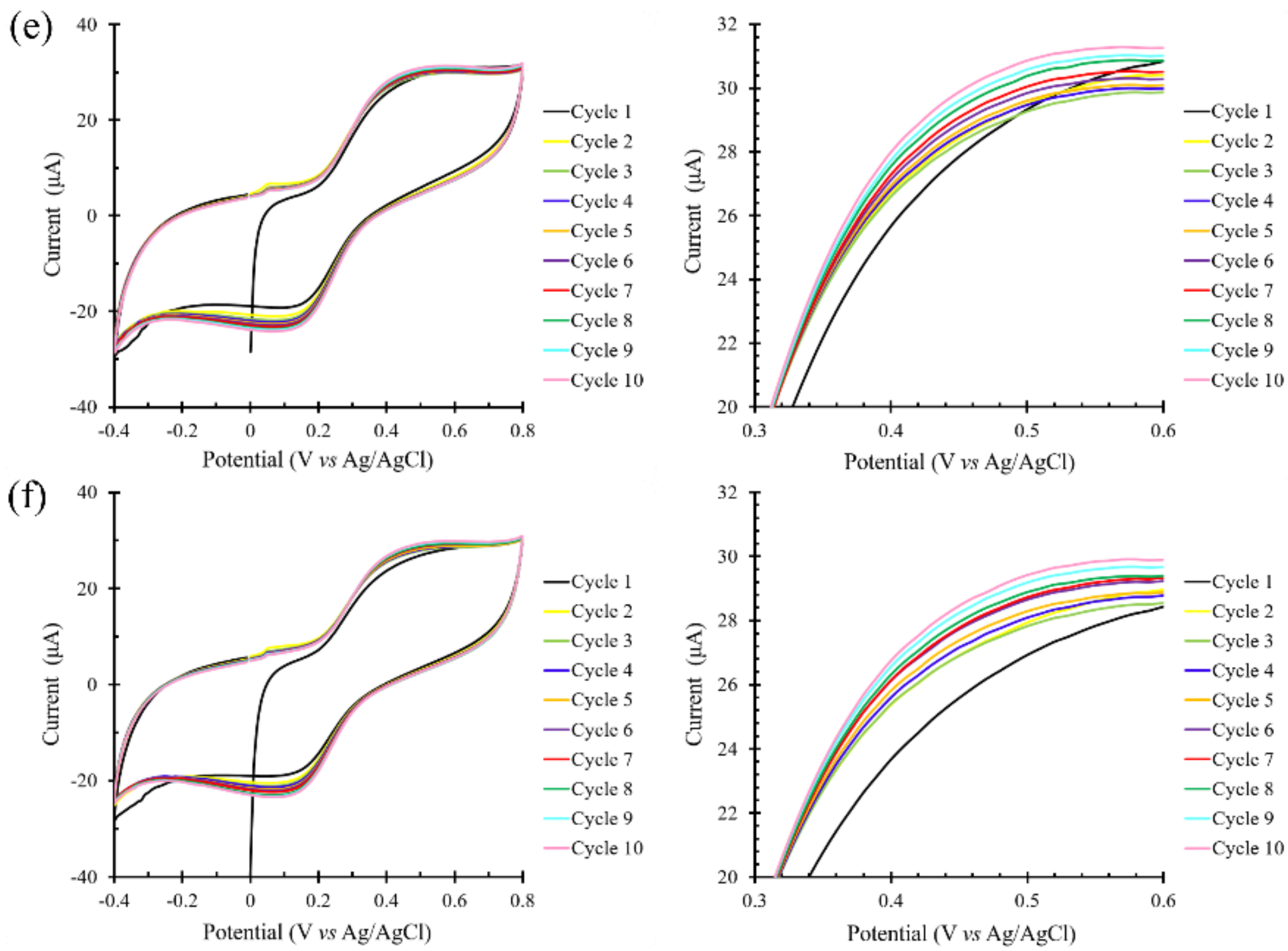

Figure S1. Cyclic voltammetric characterization. (a) Unmodified electrode (SPCE). (b) Nafion-modified electrode (SPCE/Naf). (c-f) Electrodes modified with Nafion and immobilized cardiolipin (SPCE/Naf/CL) at each antigen solution concentration: (c) SPCE/Naf/CL3, (d) SPCE/Naf/CL6, (e) SPCE/Naf/CL9, and (f) SPCE/Naf/CL12. On the left, the 10 cycles performed are shown, while on the right, an enlarged view highlights the progressive increase in peak current over the successive cycles.

Table S1. Post hoc comparisons between positive and negative control materials according to cardiolipin concentration and chronoamperometric measurement. Mean differences were calculated by subtracting the mean current obtained for the negative control material from that obtained for the positive control material (Positive – Negative) and are reported with their corresponding 95% confidence intervals (95% CI). Negative mean differences therefore indicate lower currents for the positive control material, whereas positive values indicate higher currents for the positive control material. *p*-values were adjusted for multiple comparisons using the Holm procedure. Statistical significance was established at $p < 0.05$.

**Cardiolipin concentration**	**Measurement**	**Mean difference (μA)**	**95% CI (μA)**	**Holm-adjusted *p*-value**
0.03%	$i_{5,1}$	-31.40	-37.89 to -24.91	<0.001
0.03%	$i_{5,2}$	+33.10	13.57 a 52.63	0.007
0.03%	$i_{5,3}$	+22.28	8.51 a 36.04	0.008
0.06%	$i_{5,1}$	-27.22	−33.70 a −20.73	<0.001
0.06%	$i_{5,2}$	+49.52	30.00 a 69.05	<0.001
0.06%	$i_{5,3}$	+47.08	33.31 a 60.84	<0.001
0.09%	$i_{5,1}$	-18.70	−25.19 a −12.21	<0.001
0.09%	$i_{5,2}$	+36.50	16.97 a 56.02	0.004
0.09%	$i_{5,3}$	+29.45	15.68 a 43.21	0.001
0.12%	$i_{5,1}$	-10.23	−16.71 a −3.74	0.008
0.12%	$i_{5,2}$	+50.37	30.84 a 69.89	<0.001
0.12%	$i_{5,3}$	+20.62	6.85 a 34.38	0.008

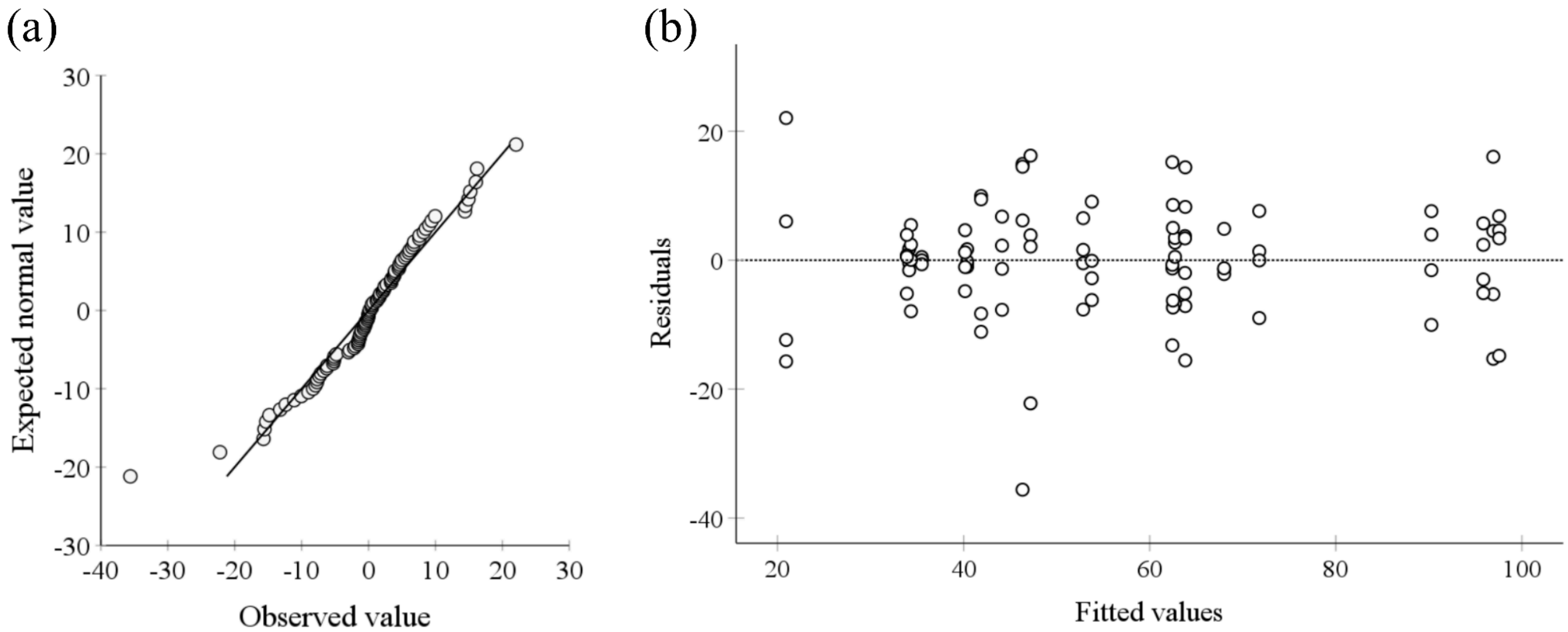

Figure S2. Diagnostic plots of the residuals from the linear mixed-effects model. (a) Normal Q–Q plot of residuals, showing the observed residual values against the expected values under a normal distribution. (b) Residuals versus fitted values plot used to assess the distribution and variance of the residuals across the range of model predictions.

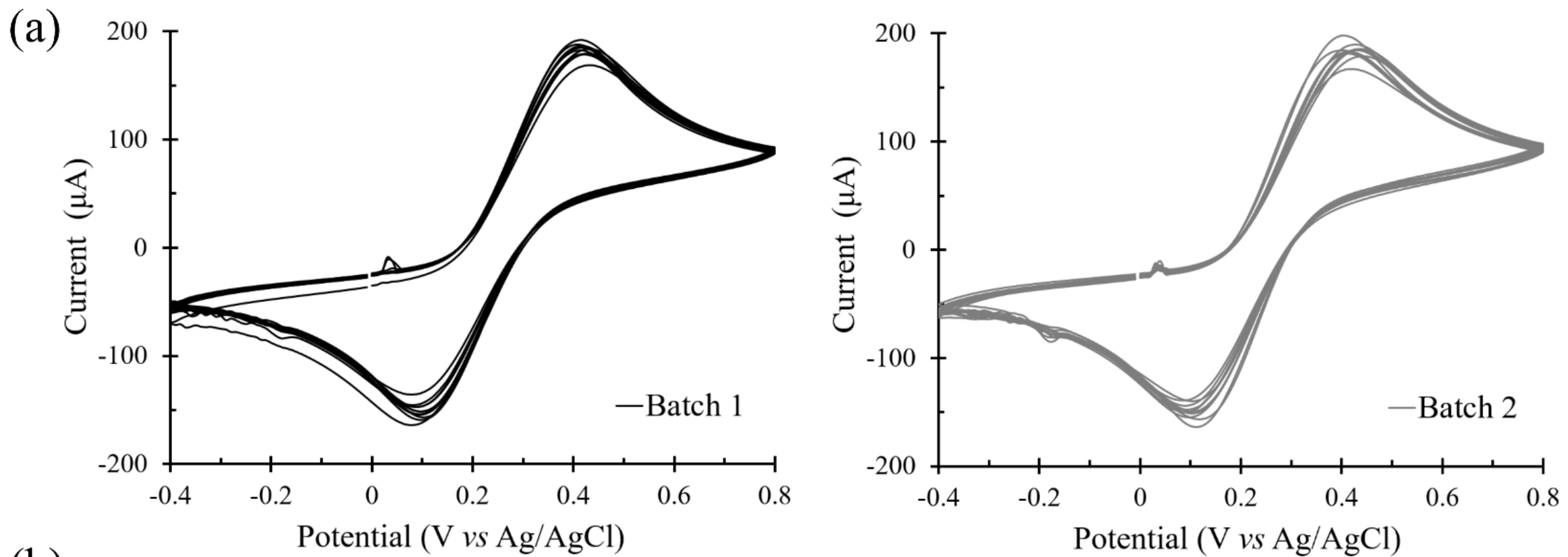

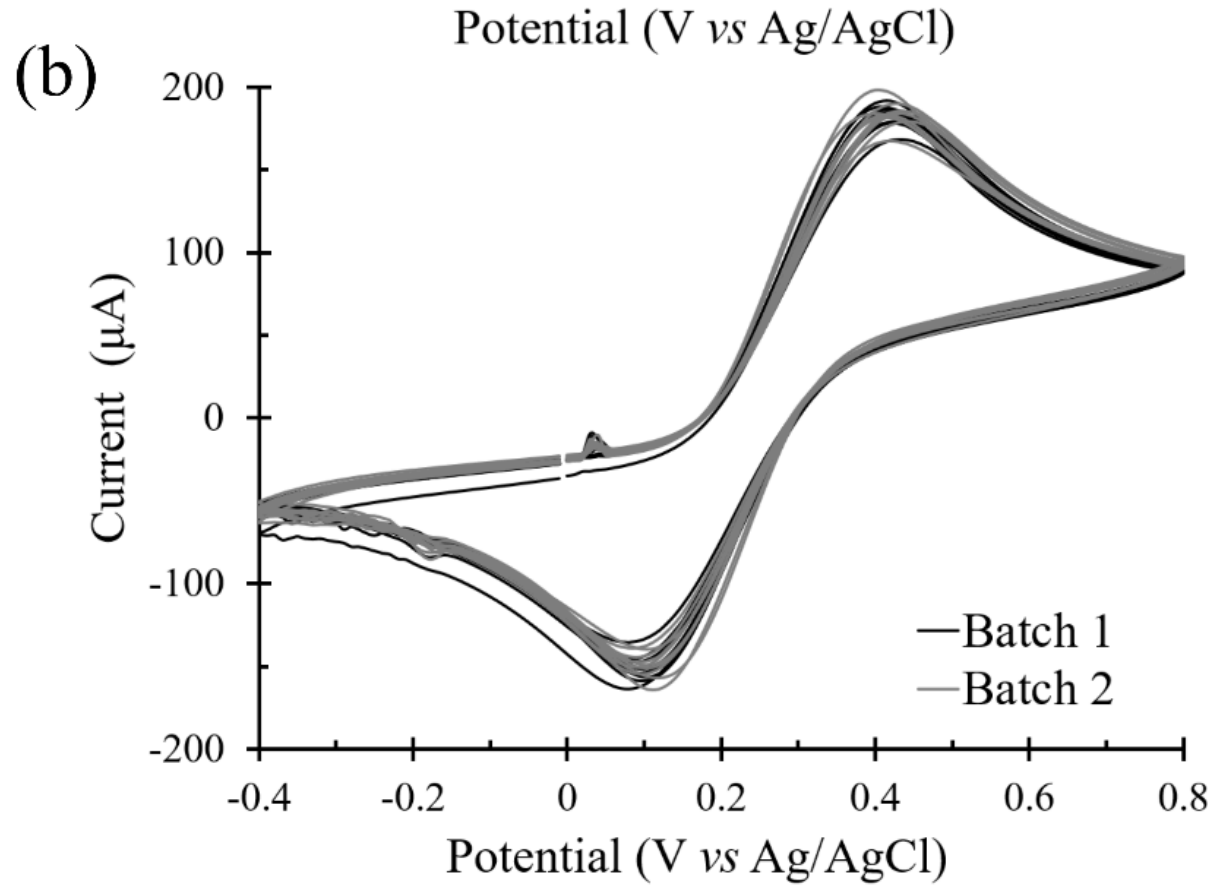

Parameter	Batch 1	Batch 2
**ipa (μA)**	182.11 ± 6.37	183.75 ± 7.93
**ipc (μA)**	-150.87 ± 7.98	-149.27 ± 7.69
**ΔE (mV)**	320.00 ± 14.91	311.00 ± 24.70
**|ipa/ipc|**	1.21 ± 0.03	1.23 ± 0.04

Figure S3. Cyclic voltammetric characterization of unmodified electrodes (SPCEs) from two different batches using 10 mM $K_4[Fe(CN)_6]$ prepared in 1 M KCl. (a) Fifth voltammetric cycles recorded for 10 SPCEs from Batch 1 (left) and 10 SPCEs from Batch 2 (right). (b) Overlay of the fifth voltammetric cycles from both batches, accompanied by the electrochemical parameters extracted from the voltammograms, including anodic peak current (ipa), cathodic peak current (ipc), peak-to-peak potential separation (ΔE), and absolute value of the peak current ratio (|ipa/ipc|). Electrochemical parameters are presented as mean ± standard deviation (n = 10 electrodes per batch).

Table S2. Two-way factorial ANOVA of the effects of control material and electrode immobilization on the electrochemical response. The current recorded at 5 s was used as the dependent variable, with control material (positive and negative) and electrode immobilization (SPCE/Naf/CL6 and SPCE/Naf) included as fixed factors. The table presents the degrees of freedom (df), F-statistics, p-values, and partial eta-squared (η^2) for the main effects and their interaction. Statistical significance was established at $p < 0.05$.

**Effect**	**df**	**F**	**p-value**	**Partial η^2**
Control material	1, 12	46.974	<0.001	0.797
Electrode immobilization	1, 12	24.790	<0.001	0.674

Table S3. Simple-effect comparisons of control material and electrode immobilization on the electrochemical response. Mean differences were calculated by subtracting the mean current recorded at 5 s for the second condition from that obtained for the first condition and are reported with their corresponding 95% confidence intervals (95% CI). The "Within" column indicates the electrode immobilization within which positive and negative control materials were compared or the control material within which electrode immobilizations were compared. Statistical significance was established at $p < 0.05$.

**Within**	**Comparison**	**Mean difference (µA)**	**95% CI (µA)**	**p-value**
SPCE/Naf/CL6	Positive vs Negative	−12.11	−14.75 to −9.47	<0.001
SPCE/Naf	Positive vs Negative	−7.69	−14.25 to −1.13	0.028
Positive	SPCE/Naf/CL6 vs SPCE/Naf	−9.40	−13.10 to −5.70	0.001
Negative	SPCE/Naf/CL6 vs SPCE/Naf	−4.98	−11.01 to 1.04	0.089

Table S4. Two-way factorial ANOVA of the effects of control material and glucose condition on the electrochemical response. The current recorded at 5 s was used as the dependent variable, with control material (positive and negative) and glucose condition (without interferent, physiological condition at 99 mg dL^{-1}, and pathological condition at 200 mg dL^{-1}) included as fixed factors. The table presents the degrees of freedom (df), F-statistics, p-values, and partial eta-squared (η^2) for the main effects. Statistical significance was established at $p < 0.05$.

**Effect**	**df**	**F**	**p**	**Partial η^2**
Control material	1, 18	270.579	<0.001	0.938
Glucose condition	2, 18	16.001	<0.001	0.640

Table S5. Pairwise comparisons between positive and negative control materials within each glucose condition. Mean differences were calculated by subtracting the mean current obtained for the negative control material from that obtained for the positive control material (Positive – Negative) and are reported with their corresponding 95% confidence intervals (95% CI). *p-values* were adjusted using the Bonferroni procedure. Statistical significance was established at $p < 0.05$.

**Glucose condition**	**Mean difference (µA)**	**95% CI (µA)**	**Bonferroni-adjusted p-value**
Without interferent	−12.11	−15.10 to −9.12	<0.001
Physiological (99 mg dL^{-1})	−8.43	−11.42 to −5.45	<0.001
Pathological (200 mg dL^{-1})	−11.70	−14.68 to −8.71	<0.001

Table S6. Pairwise comparisons among glucose conditions within each control material. Mean differences were calculated by subtracting the mean current recorded at 5 s for the second condition from that obtained for the first condition and are reported with Bonferroni-adjusted 95% confidence intervals (95% CI). Comparisons were performed separately for the positive and negative control materials. *p-values* were adjusted using the Bonferroni procedure. Statistical significance was established at $p < 0.05$. WI: without interferent; Physiological: 99 mg dL^{-1}; Pathological: 200 mg dL^{-1}.

**Control material**	**Comparison**	**Mean difference (µA)**	**95% CI (µA)**	**Bonferroni-adjusted *p-valu*e**
Positive	WI vs Physiological	+2.50	−0.49 to 5.49	0.121
Positive	WI vs Pathological	+3.09	0.10 to 6.08	0.041
Positive	Physiological vs Pathological	+0.59	−2.40 to 3.58	>0.999
Negative	WI vs Physiological	+6.18	3.19 to 9.17	<0.001
Negative	WI vs Pathological	+3.50	0.51 to 6.49	0.019
Negative	Physiological vs Pathological	−2.68	−5.67 to 0.31	0.088
